# Human Transbodies to HCV NS3/4A Protease Inhibit Viral Replication and Restore Host Innate Immunity

**DOI:** 10.3389/fimmu.2016.00318

**Published:** 2016-08-26

**Authors:** Surasak Jittavisutthikul, Watee Seesuay, Jeeraphong Thanongsaksrikul, Kanyarat Thueng-in, Potjanee Srimanote, Rolf G. Werner, Wanpen Chaicumpa

**Affiliations:** ^1^Graduate Program in Immunology, Department of Immunology, Faculty of Medicine Siriraj Hospital, Mahidol University, Bangkok, Thailand; ^2^Department of Parasitology, Faculty of Medicine Siriraj Hospital, Center of Research Excellence on Therapeutic Proteins and Antibody Engineering, Mahidol University, Bangkok, Thailand; ^3^Graduate Program in Biomedical Science, Faculty of Allied Health Sciences, Thammasat University, Pathum-thani, Thailand; ^4^School of Pathology, Institute of Medicine, Suranaree University of Technology, Nakhon Ratchasima Province, Thailand; ^5^Industrial Technology, Faculty of Science, University of Tuebingen, Tuebingen, Germany

**Keywords:** cell-penetrating antibody (transbody), hepatitis C virus, innate immunity, phage display, NS3/4A protease, qRT-PCR, single chain antibody

## Abstract

A safe and effective direct acting anti-hepatitis C virus (HCV) agent is still needed. In this study, human single chain variable fragments of antibody (scFvs) that bound to HCV NS3/4A protein were produced by phage display technology. The engineered scFvs were linked to nonaarginines (R9) for making them cell penetrable. HCV-RNA-transfected Huh7 cells treated with the transbodies produced from four different transformed *E. coli* clones had reduced HCV-RNA inside the cells and in the cell spent media, as well as fewer HCV foci in the cell monolayer compared to the transfected cells in culture medium alone. The transbodies-treated transfected cells also had up-expression of the genes coding for the host innate immune response, including *TRIF, TRAF3, IRF3, IL-28B*, and *IFN-β*. Computerized homology modeling and intermolecular docking predicted that the effective transbodies interacted with several critical residues of the NS3/4A protease, including those that form catalytic triads, oxyanion loop, and S1 and S6 pockets, as well as a zinc-binding site. Although insight into molecular mechanisms of the transbodies need further laboratory investigation, it can be deduced from the current data that the transbodies blocked the HCV NS3/4A protease activities, leading to the HCV replication inhibition and restoration of the virally suppressed host innate immunity. The engineered antibodies should be tested further for treatment of HCV infection either alone, in combination with current therapeutics, or in a mixture with their cognates specific to other HCV proteins.

## Introduction

Before 2011, patients with hepatitis C virus (HCV) infection were weekly injected with pegylated-interferon-alpha combined with daily oral ribavirin (a nucleoside analog), which is a standard-of-care (SOC). The treatment conferred high rate of sustained virologic response (SVR) against HCV of most genotypes by restoring the virally suppressed host innate immunity and restraining the viral load (1). However, HCV strains of genotype 1 were relatively refractory to the SOC. Besides, the regimen is prolonged, stringent, and costly, as well as induces severe adverse effects, which are poorly tolerated by a significant fraction of the recipients. During the past decade, intense research has focused on development of direct acting anti-HCV agents (DAAs) that interfere with the functions of several HCV proteins. These are, for examples, inhibitors of NS3/4A protease (e.g., telaprevir, boceprevir, simeprevir, asunaprevir, danoprevir, faldaprevir, paritaprevir); NS5B polymerase (e.g., sofosbuvir, dasabuvir); a protein important for membrane web formation, NS4B [e.g., substituted imidazo(1,2-a) pyrimidines, clemizole, piperazinones]; a regulatory phosphoprotein of the viral replication complex, NS5A (e.g., ombitasvir, daclatasvir, ledipasvir, velpatasvir); or capsid protein (2–10). Also, drugs that inhibit HCV–host protein interaction have been tested (11, 12). Presently, several DAAs have received regulatory approval for clinical use, such as protease inhibitors (boceprevir, telaprevir, and semiprevir), polymerase inhibitor (sofosbuvir), and NS5A inhibitor (ledipasvir). Additional DAAs are in various stages of clinical development (9, 13). Many new drug regimens, when administered either singly, together with SOC, or in various combinations among themselves, conferred improved SVR rate compared with SOC (9, 13–16). However, the challenges that the new drugs are facing include complicated treatment protocols, additional severe side effects, drug–drug interaction, and emergence of drug- and cross-drug-resistant HCV mutants. The new regimens are contraindicated for some groups of patients/subjects (16–21). Thus, safe and effective DAAs are needed.

Passive immunization (administration of ready-made antibody) has been practiced for prevention/intervention and treatment of diseases since the late eighteenth century (called serum therapy/serotherapy at the time) (22). Nevertheless, the physicochemical properties of the plasma membrane impede accessibility of antibodies to their intracellular targets (such as proteins/enzymes of replicating virus). To circumvent this obstacle, several peptides called cell-penetrating peptides (CPPs) have been developed for delivering antibodies/antibody fragments and a variety of other cargoes (proteins, drugs, nucleic acids, plasmids, and siRNAs) across the plasma membrane into cytosol and also to different subcellular compartments (23–27). Examples of the CPPs are protein transduction domains (such as penetratin, HIV-1 Tat peptide) (28), amphipathic peptides such as protein-rich motif (SAP) (29), and other CPP type, including nonaarginine (R9) and polylysine (30). In our laboratory, cell-penetrable antibodies (transbodies) specific to viral proteins have been prepared by linking the antibody molecules to either penetratin or R9. These fusion proteins readily entered mammalian cells and bound to their respective intracellular targets (31–35).

Usually, an antibody molecule uses several amino acids in the complementarity determining regions (CDRs) to interact with multiple residues/sites of the target, which makes it difficult for the pathogens to escape the antibody binding. On the contrary, pathogens may resist small molecular inhibitors through a single amino acid mutation (21). In this study, cell-penetrable human single chain antibody variable fragments (transbodies) to HCV protease that inhibit the viral replication and restore the host innate immune response were produced. It is envisaged that a right combination of cell-penetrable human/humanized antibodies that target pivotal enzymes of the HCV, i.e., helicase (31), polymerase (32, 33), and protease (34), a strategy similar to the precedent anti-HIV regimen, should be a novel anti-HCV remedy that is safe, genotype independent, and more tolerable to the viral mutation.

## Materials and Methods

### Recombinant HCV NS3/4A Fusion Protein Production

The recombinant NS3/4A protein (rNS3/4A) with the HCV-inherent serine protease activity was produced from BL21 (DE3) *E. coli* carrying pET23b^+^-*NS3/4A*, as described previously (34). The fusion protein contained 180 amino acids of the *N*-terminal one-third of the NS3 of the genotype 3a HCV linked with residues 21–32 of the NS4A of the same genotype. The recombinant NS3/4A protein cleaved a fluorescent peptide substrate that contained nine amino acids at the protease cleavage site as determined by a continuous fluorescence resonance energy transfer (FRET) assay (34).

### Mouse Anti-NS3/4A Polyclonal Antibody

The experiments received approval from the Siriraj Bio-safety Risk Management Taskforce (SI 2014-004). Three ICR mice (National Laboratory Animal Center, Mahidol University, Nakhonpathom, Thailand) were housed in a shoe-box typed cage under 12/12 light/dark cycle at 23–25°C and 55–60% humidity. Feed and water were supplied *ad libitum*. The animals were immunized intraperitoneally with three doses at 2-week intervals of 10 μg purified recombinant NS3/4A protein mixed with alum (Pierce, USA) at ratio 1:3. After the last booster, the mice were bled and their serum titers were assessed against the homologous antigen by indirect ELISA.

### Immunological Methods

Indirect ELISA was performed as described previously (33) using 1 μg of purified rNS3/4A protein in 100 μl of carbonate–bicarbonate buffer, pH 9.6 to coat each well of an ELISA plate. Five percent skim milk in PBS (300 μl/well) was used for blocking the well surface before adding antibody preparations (e.g., serial dilutions of the mouse immune sera, HuscFv preparations) into appropriate wells. Antibody control was either normal mouse serum or lysate of original *E. coli*. Bovine serum albumin (BSA) served as control antigen and PBS as blank. After incubation, all wells were washed with the PBS containing 0.05% Tween-20 (PBST). Horseradish peroxidase (HRP)-conjugated anti-immunoglobulin isotype (Southern Biotech, Birmingham, AL, USA) and chromogenic substrate (KPL, Gaitherburg, MD, USA) were used for color development. The enzymatic reaction was stopped by adding 1% SDS. OD_405nm_ of contents in all wells were determined against blank.

For SDS-PAGE and Western blot analysis (WB), polyacrylamide gel at a desired gel percentage cast in a Mini-PROTEAN-3^®^ Cell System (Bio-Rad, Hercules, CA, USA) was used. Samples prepared in 6 × SDS gel reducing buffer were boiled for 10 min before loading into the gel slots made in the 4% stacking gel. PageRuler^®^ pre-stained protein ladders (Fermentas, Thermo Fisher Scientific, MA, USA) were included in one or two gel slots as molecular weight marker. Electrophoresis was carried out in Tris–glycine running buffer, pH 8.3, initially at 10 mA per gel until the bromphenol blue dye font reached the resolving gel; then the electricity was increased to 20 mA and continued until the dye font reached the lower gel edge. Separated proteins in the gel were either revealed by staining with Coomassie Brilliant Blue G-250 dye or they were electroblotted onto nitrocellulose membrane (NC) for WB. For WB, the blotted NC was blocked either with 3% BSA or 5% skim milk in PBS. The blocked NC was then probed with antibody preparation; then the antigen–antibody reactive bands were revealed using alkaline phosphatase (AP)-labeled anti-isotype antibody and AP chromogenic substrate.

### Phage Clones That Bound to the rNS3/4A Protein and the Phage Transformed *E. coli* Clones

Phage clones bound to the rNS3/4A fusion protein were fished-out from a human scFv phage display library as described previously (33) using purified rNS3/4A (1 μg) as bait. The rNS3/4A-bound phages were put into HB2151 *E. coli* bacteria, and the bacteria were spread on a 2 × YT-AG plate. Direct colony PCR (36) was used to screen *E. coli* colonies that carried HuscFv coding genes (*huscfvs*). Lysates of *huscfv*-positive *E. coli* colonies grown in 0.5 mM IPTG-conditioned broth were determined by Western blotting for the presence of the E-tagged-HuscFvs.

### Characterization of the Bacterially Derived-HuscFvs

Spectrometrically standardized soluble HuscFvs in the lysates of transformed HB2151 *E. coli* clones were tested for binding to the rNS3/4A by indirect ELISA and Western blotting. Original HB2151 *E. coli* (HB) was used as background binding control in both assays. BSA served as control antigen in the indirect ELISA.

Diversity of the *huscfvs* sequences of the HB2151 *E. coli* clones were determined by subjecting the PCR amplified *huscfvs* to *Mva*I digestion followed by resolving the cut DNA products in 14% polyacrylamide gel containing 0.5% glycerol. Electrophoresis was carried out in TBE buffer at 20 mA per slab gel. The separated DNA bands were stained by ethidium bromide and the restriction fragment length polymorphism (RFLP) was determined using a Bio Doc-ITTM Imaging System, UVP, USA. CDRs and their respective canonical immunoglobulin framework regions (FRs) of the sequenced *huscfvs* were predicted using an online Internatioanl ImMunoGeneTics (IMGT^®^) Information System.

### Generation of Cell Penetrating HuscFvs (Transbodies)

Because the antibodies must bind to the target inside the HCV-infected cells, they were linked to nonaarginine (R9), which is a cell-penetrating peptide, as follow: the *huscfvs* were amplified from the pCANTAB5E phagemids using a Q5 High Fidelity DNA polymerase (Thermo Fisher Scientific). The specific primers were forward-*R9-huscfv-LIC*: 5′-GGTTGGGAATTGCAACGTCGCCGTCGCTCGCCGTCGCCGTCGCCGTGCGGCCCAGCCCGGCC-3′ and reverse-E-tag-LIC: 5′-GGAGATGGGAAGTCATTAACGCGGTTCCAGCGGATCC-3′. The reaction mixture contained 15.75 μl sterile distilled water, 5.0 μl Q5 reaction buffer (5×), 0.5 μl dNTP mix (10 mM each), forward and reverse primers (10 μM in 1.25 μl each), 1.0 μl of *huscfv*-phagemid template (3 ng DNA template), and 0.25 unit of Q5 Hotstart DNA polymerase. The thermal cycles were first denaturation at 98°C, 30 s; 34 cycles of denaturation at 98°C for 30 s, annealing/extension at 69°C for 10s, and final extension at 72°C, 5 min; and hold at 4°C, several min. The PCR product was stained with ethidium bromide, verified, and purified from agarose gel using DNA extraction kit (Jena Bioscience, Jena, Germany). The 5′- and 3′- overhangs of the R9-*huscfv* amplicons were generated by setting up the following reaction mixtures: 9 μl of sterile distilled water, 2 μl of 5× LIC buffer, 0.1 pmol of the purified PCR product, and 1 μl T4 DNA polymerase and dGTP. The reaction mixtures were kept at 25°C for 5 min and stopped by adding 0.6 μl of 0.5 M EDTA. Annealing of the DNA products with 15 base pairs (bp) 5′-overhang to the pLATE52 vector containing complementary overhang was performed by mixing 1 μl of the vector and 60 ng (0.02 pmol) LIC ready vector (Thermo Fisher Scientific). The mixtures were kept at 25°C for 5 min before putting into JM109 *E. coli* by heat-shock method. The transformed *E. coli* clones carrying the recombinant pLATE52-*R9-huscfv* plasmids were screened by PCR using the pLATE52 specific primers, i.e., LIC forward sequence: 5′-TAATACGACTCACTATAGGG-3′ and LIC reverse sequence: 5′-GAGCGGATAACAATTTCACACAGG-3′. The PCR reaction mixture was: 12.7 μl sterile distilled water, 2 μl PCR buffer + KCl (10×), 1.2 μl 25 mM MgCl_2_, 2 μl dNTP mix (10 μM each), 1 μl (10 μM) each of LIC primers, and 0.5 unit of *Taq* polymerase.

The pLATE52-*R9-huscfv* were extracted from the PCR positive clones, purified, put in Rosetta™2 (DE3)-competent cells (Novagen, Schwalbach, Germany), and spread onto selective LB agar plates supplemented with 100 μg/ml ampicillin and 34 μg/ml chloramphenicol (Calbiotech, Spring Valley, CA, USA) (LB-AC agar). The sibling *E. coli* colonies grown on the agar plates were randomly picked and screened for the presence of the pLATE52-*R9-huscfv* plasmids by PCR using the pLATE52 primers. The Rosetta™2 (DE3) bacteria with the pLATE52-*R9-huscfv* plasmids were cultured in 0.5 mM IPTG-conditioned broth. The induced bacterial cells were collected and R9-HuscFvs in their homogenates were determined by SDS-PAGE and Western blotting using anti-6× His as the R9-HuscFv-detection reagent. The clones that expressed the R9-HuscFvs (with 6× His and T7 tags at the *N*-terminal and E-tag at the *C*-terminal) were kept as stocks in 20% glycerol at −80°C until use.

### Large-scale Production, Purification, and Refolding of the Cell Penetrating HuscFvs

The Rosetta™2 (DE3) *E. coli* clones carrying the pLATE52-*R9-huscfv* were grown in 2× YT-AC broth at 37°C with shaking at 250 rpm for 16 h. Ten milliliters of the overnight culture were inoculated into 250 ml of 2× YT-AC broth in a 2-liter flask and incubated with shaking aeration at 37°C until OD_600nm_ was approximately 0.8–0.9 (~3 h). The culture was added with IPTG (final concentration of 1 mM), incubated at 30°C for 6 h, and centrifuged at 5,000 × *g* at 4°C for 20 min. To prepare the bacterial inclusion bodies (IBs), each 2 g of the *E. coli* wet pellets were lysed with 10 ml of BugBuster™ protein extraction reagent (Novagen, Schwalbach, Germany) and 20 μl of Lysonase™ bioprocessing reagent (Novagen). The preparation was kept at 25°C on a rotator for 20 min and centrifuged at 8,000 × *g* at 4°C for 30 min. IB in the pellet was washed twice with Wash-100 reagent and once with Wash-114 reagent with shaking at high speed for 40 min and centrifuged. The IB was then washed with Wash-Solvent reagent and Milli Q water on ice also with vigorous shaking and centrifuged.

For HuscFv refolding, 5 ml of buffer [50 mM CAPS, pH 11.0; 0.3% (w/v) *N*-Lauryl sarcosine; and 1 mM DTT] were added to reconstitute 5 mg of purified IB and kept at 4°C for 16 h. After dissolving completely, the protein was loaded into the Slide-A-Lyzer^®^ 2K Dialysis Cassettes G2 (Thermo Fisher Scientific), and the cassette was subjected to dialysis against 750 ml of a refolding buffer (20 mM imidazole, pH 8.5, supplemented with 0.1 mM DTT) at 4°C with slow stirring. After 3 h, the buffer was changed to fresh refolding buffer, and the dialysis was continued for 16 h. The refolded protein was subsequently dialyzed against a dialysis buffer (20 mM imidazole without DTT) with slow stirring at 4°C for 16 h. The preparation was filtered through a 0.2-μm low protein binding Acrodisc^®^ Syringe Filter (Pall, NY, USA) and kept in 30°C water-bath for additional 2 h before adding with 60 mM trehalose. Protein concentration of the refolded HuscFvs was measured (Pierce^®^ BCA Protein Assay), while quality and purity of the proteins were determined by SDS-PAGE and protein staining. The antibodies were tested for binding to the homologous antigen (rNS3/4A) by indirect ELISA.

### Cell Penetrating Ability of the R9-HuscFvs, Their Intracellular Target Binding, and Toxicity

The experiments involving HCV of this study received approval from the Siriraj Bio-safety Risk Management Taskforce. The pJFH-1 (GenBank AB047639), which carried a full-length HCV genotype 2a cDNA, was used for production of HCV RNA, as described previously (32, 33). Briefly, the plasmid was cut with *Xba*I endonuclease and transcribed using a T7-transcription kit (MEGAscript^®^, Ambion, Foster City, CA, USA). Ten micrograms of JFH-1 RNA were put in Huh7 cells (4 × 10^6^ cells) suspended in 0.4 ml of reduced serum medium (Opti-MEM, Invitrogen, USA) by means of electroporation; complete DMEM containing 10% heat-inactivated fetal bovine serum (Hyclone, USA) and antibiotics was immediately added to the electroporated cells, which were then distributed onto individual cover slips (2 × 10^4^ cells/slip) placed in wells of a 24-well tissue culture plate After incubating at 37°C in 5% CO_2_ incubator overnight and rinsed with sterile PBS, the cells were replenished with 500 μl complete DMEM containing 10 μg of individual R9-HuscFvs. After 1 h, culture supernatants were removed; the cells were washed with PBS, fixed with 1% Triton X-100, 30 min; blocked with 3% BSA; and washed. Rabbit anti-6× His polyclonal antibody (Abcam, Cambridge, UK) (1:500) was added to the cells and incubated at 37°C for 1 h, washed, and 1:1000 mouse anti-NS3/4A polyclonal antibody was added. After 1 h, the cells were washed and added with goat anti-rabbit Alexa Flour 488 (1:500) and donkey anti-mouse Alexa Flour^®^647 (Abcam). DAPI (1:5000) (Invitrogen, Carlsbad, CA, USA) was used to stain nuclei. The preparations were kept at 37°C for 1 h, and the stained cells were observed under a confocal microscope.

CytoTox96^®^ non-radioactive cytotoxicity (lactate dehydrogenase, LDH) assay kit (Promega, Madison, WI, USA) and Cell Titer-Glo^®^ Luminescent Cell Viability Assay kit (Promega) were used for determining cytotoxicity of the R9-HuscFvs to Huh7 cells. The LDH release assay was performed as described previously (35). Huh7 monolayer in individual wells of a 96-well tissue culture plate (2 × 10^4^ cells/well) were washed with sterile PBS, added with 25 μg of R9-HuscFvs in complete DMEM, and incubated for 24 h at 37°C in 5% CO_2_ atmosphere. Controls were cells in the medium alone (minimal/spontaneous release) and cells added with 10% SDS (maximal release) were included. After 24 h, culture supernatants (triplicate wells of each treatment) were collected separately. Fifty microliters of culture fluid were mixed with the assay buffer provided with the kit and kept at 25°C for 30 min under light protection. The reactions were stopped by adding 1 M acetic acid, and OD_490nm_ was determined. Percent cytotoxicity was (LDH release of test − spontaneous LDH release)/(Maximal LDH release − spontaneous LDH release) × 100 (35). Percent cell viability was calculated by subtracting 100 with percent cytotoxicity.

For the Luminescent Cell Viability Assay that quantifies the number of viable cells based on the amounts of ATP released by metabolically active cells, Huh7 cells cultured in complete DMEM were seeded into wells of a 96-well opaque tissue culture plates (Corning) (2 × 10^4^ cells/well). After incubating at 37°C in 5% CO_2_ atmosphere for 24 h, the spent media were removed, and the tested cells were added with 25 μg of purified R9-HuscFvs in 100 μl complete DMEM. Medium only was added to the control cells (negative control). After keeping at 37°C in 5% CO_2_ incubator for 48 h, the plate was brought to room temperature (25°C) for 30 min; the medium in each well was replaced with 100 μl of the Glo reagent, kept on an orbital shaker for 2 min, then at room temperature for 10 more min for luminescence stabilization. The luminescence signal of each well was recorded using Synergy™ H1 Multi-mode Reader (BioTek^®^). Luminescence of the well containing medium only served as background luminescence. The percentage of cell viability was (Luminescence of test − Background luminescence)/(Luminescence of negative control − Background luminescence) × 100.

For *in vivo* toxicity testing of the R9-HuscFv, a protocol of Thai Pharmacopia was followed. Two groups of male BALB/c mice (6–8 weeks old) were used. Each mouse of group 1 (*n* = 5) was injected intraperitoneally with 25 μg of R9-HuscFv/g body weight in 200 μl buffer. This antibody dose is higher than those used for passive immunization of human (37). Three more booster doses were given on every alternate day. Control mice (group 2; *n* = 4) were injected with buffer alone. The animals were observed daily for any sign of morbidity, including ruffled hair, eye and nasal discharge, diarrhea, loss of appetite, and body temperature. Their body weights were determined also. Seven days post-last booster, all mice were bled and their sera were monitored for some parameters of blood chemistry including liver enzymes (AP, aspartate aminotransferase, and alanine aminotransferase) and total bilirubin.

### Transbody-mediated Inhibition of the HCV Replication

Huh7 cells transfected with JFH-1 RNA was prepared as above. Aliquots of the transfected cells (2 × 10^5^ cells/well) were added to individual wells of a 12-well tissue culture plates and kept for 3 h at 37°C in 5% CO_2_ incubator. After washing with sterile PBS, the cells were added with 25 μg of purified R9-HuscFvs dissolved in complete DMEM. Negative and background inhibition controls were the infected cells added with medium alone and medium containing 25 μg of control R9-HuscFv, respectively. Positive inhibition controls were 100 units of pegylated (PEG) interferon + 250 μM ribavirin, 0.175 μM telaprevir (VX-950) (Selleckem, Houston, TX, USA), and PEG-interferon + 250 μM ribavirin + 0.175 μM telaprevir. After 5 more days of incubation, total RNAs were extracted from the cells and their spent media (Trizol™ reagent, Invitrogen). Amounts of HCV 5′ UTR in all samples were quantified by qRT-PCR (33). Alternatively, culture supernatants of the infected cells in all wells were removed, and the viral foci formed in the respective cell monolayer were enumerated after staining for the HCV core protein (33).

### Quantitative Real-time RT-PCR

The qRT-PCR reaction mixture (12.5 μl) was prepared: 6.25 μl 2× Brilliant II SYBR^®^ Green qRT-PCR master mix, 0.5 μl each of the JFH-1 5′ UTR primers, 0.5 μl RT/RNS block enzyme, RNA template up to 4.75 μl (200–900 ng of standard RNA), and sterile DEPC-treated distilled water to 12.5 μl. The amplification was carried out using Mx3000P QPCR System (Agilent Technologies) at 42°C for 30 min, 55°C for 30 min, initial denaturation at 95°C for 10 min, and 40 cycles of 95°C for 30 s, 60°C for 30 s, and 72°C for 30 s. To analyze the dissociation curve, a thermal profile of 95°C for 1 min, ramped down to 55°C for 45 s at a speed of 0.5°C/s and ramped up to 95°C was carried out. C_t_ of 10-fold serially diluted pJFH-1 with full-length HCV genotype 2a cDNA insert, calculated 2.77 × 10^7^ to 0.02 copy numbers, were used for constructing a standard curve. Log_10_ RNA copies/ml of sample was extrapolated from the standard curve. Data of one of the three independent and reproducible experiments are reported herein.

### HCV Foci Assay

Hepatitis C virus foci formed in the JFH-RNA-transfected Huh7 cell monolayer after receiving different treatments were enumerated (34). Briefly, the spent culture fluids of the transfected cells of all treatments were discarded. After washing, the cells were fixed with absolute methanol at 4°C, 15 min; blocked for 1 h with 10% BSA at 37°C; incubated with mouse anti-HCV core polyclonal antibody; washed; added with AP-conjugated-goat anti-mouse isotype; washed again; and added with AP chromogenic substrate (KPL). Untreated transfected cells and non-transfected (normal) cells were included in the experiments as negative inhibition (maximal number of HCV foci) and negative foci control, respectively. Numbers of HCV foci in each well were counted under 40× magnification using an inverted fluorescence microscope (NIS-Element D version 4.10.0.8310 W/camera, Ti-S Intensilight Ril NIS-D, Nikon, Japan).

### Response of the HCV-RNA-transfected Cells after Treatment with the Transbodies and Controls

After treating the JFH-1 RNA-infected Huh7 cells with various R9-HuscFvs and controls (control R9-HuscFv, PEG-IFN + ribavirin, telaprevir, PEG-IFN + ribavirin + telaprevir, and medium alone), expressions of innate immune response genes (amounts of mRNAs), including *TRIF, TRAF3, IRF3, IL-28B*, and *IFNβ*, were determined by qRT-PCR as described previously (34) using GAPDH mRNA for normalization.

### Computerized Simulation for Predicting Residues of the HCV NS3/4A Protein Bound by the HuscFvs

Three-dimensional structure of NS3/4A (PDB ID-1A1R, RCSB database) was retrieved. Tertiary structures of HuscFvs were modeled by subjecting their amino acid sequences to the I-TASSER server (38). The I-TASSER-predicted models were subsequently refined and improved to their nearest native state (39, 40). Molecular docking between the NS3/4A 3D and the HuscFv models was performed by ClusPro2.0 server (41). Pymol software (The PyMOL Molecular Graphics System, version 1.3r1 edu, Schrodinger, LLC) was used to build and visualize the protein complexes that formed the largest cluster size and the minimal local energy.

### Statistical Analysis

Means and SDs of tests and controls were compared using unpaired *t*-test. *p* < 0.05 was considered significantly different.

## Results

### Phage-transformed *E. coli* Clones That Produced HCV NS3/4A-bound HuscFvs and the HuscFv Characteristics

**Figure 1:**
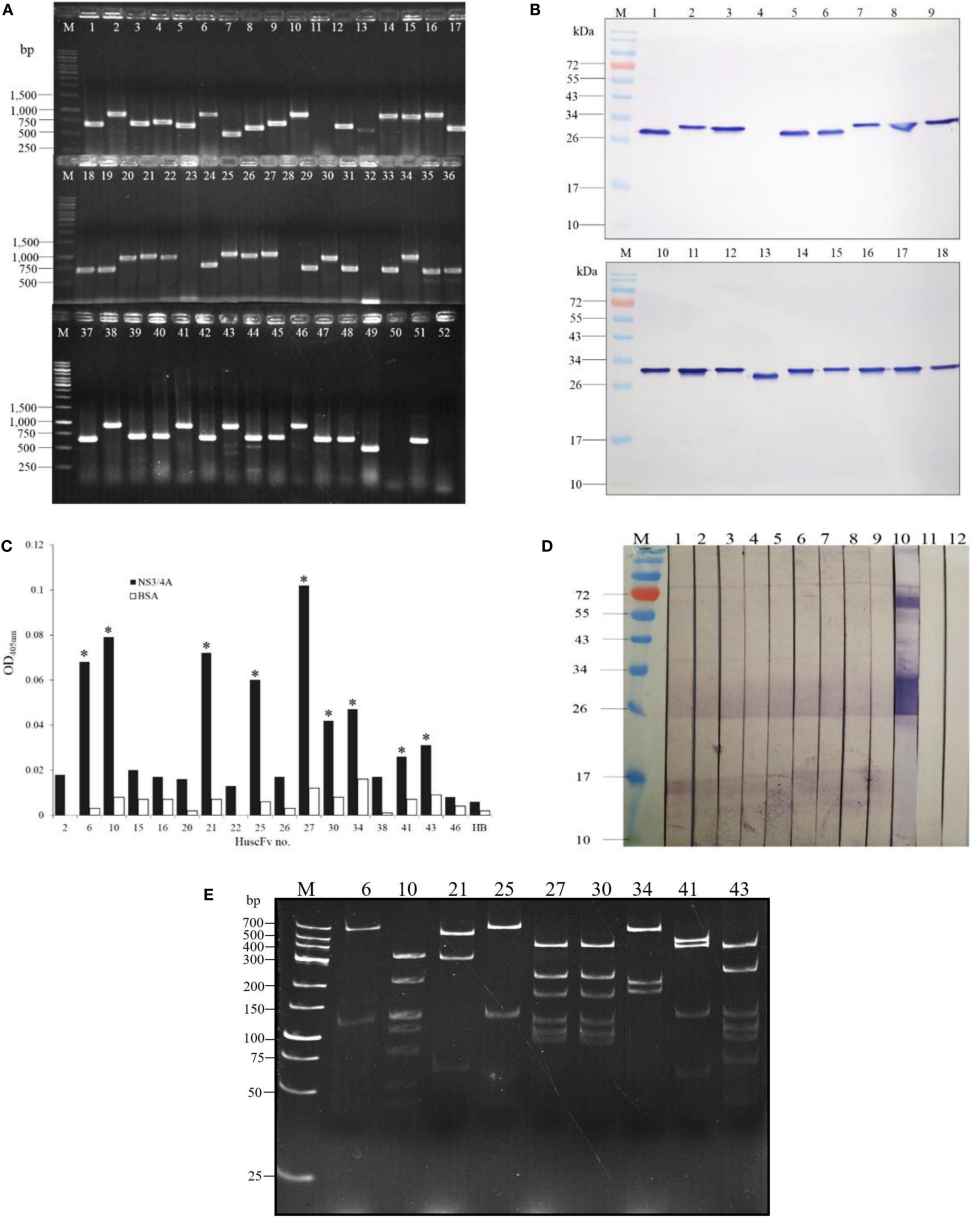
**Preparation of *E. coli* clones that expressed NS3/4A-bound HuscFvs**. Results of PCR for amplification of *huscfvs* using 52 *E. coli* bacteria infected with individual phages that bound to recombinant NS3/4A as templates; 18 clones (34.6%) revealed the *huscfvs* at about 1,000 base pairs (bp) **(A)**. After growing the *huscfv*-positive clones under 0.5 mM IPTG induction, the bacterial lysates were checked for HuscFvs by Western blot analysis. Seventeen of the 18 clones produced the HuscFvs seen as bands at ~27–32 kDa **(B)**. After standardizing spectrophotometrically, the HuscFvs were tested for their ability to bind to the NS3/4A by indirect ELISA. The antibodies produced by nine phage-transformed *E. coli* clones (nos. 6, 10, 21, 25, 27, 30, 34, 41, and 43) gave significant ELISA signal to the rNS3/4A above control antigen (BSA) and negative HuscFv contol (HB or lysate of original HB2151 *E. coli*) **(C)**. Binding of the HuscFvs from the nine clones was verified by Western blotting. The HuscFvs were used to probe the SDS-PAGE-separated rNS3/4A, and all of them bound to the antigen seen as diffuse bands at ~26–32 kDa (lanes 1–9) **(D)**; lane 10, positive binding control which the SDS-PAGE-separated rNS3/4A was probed with mouse anti-NS3/4A polyclonal antibodies. Lanes M of **(B)** and **(D)**, protein marker; numbers at the left are protein molecular masses in kDa. Restriction fragment length polymorphism (RFLP) of the *huscfvs* of the clones **(E)**. Lanes M of **(A)** and **(E)**, DNA marker; numbers at the left are DNA sizes in bp.

Fifty-two HB2151 *E. coli* bacteria infected with the rNS3/4A-bound phages that were grown on the selective LB-A agar plate were checked for the presence of *huscfvs*, and 18 clones (34.6%) revealed the genes at about 1,000 bp (Figure 1A). After growing these *E. coli* clones under 0.5 mM IPTG-conditioned broth, lysates of 17 clones contained HuscFvs seen as bands at ~27–32 kDa (Figure 1B). The HuscFvs of nine phage-transformed *E. coli* clones (nos. 6, 10, 21, 25, 27, 30, 34, 41, and 43) gave significant ELISA signal above controls (Figure 1C) and bound to SDS-PAGE separated-rNS3/4A by WB (Figure 1D). The nine clones revealed eight different *huscfv* patterns (eight RFLP types) after cutting with *Mva*I; clones no. 6, 10, 21, and 25 had patterns 1–4, respectively; clones no. 27 and 30 had the same pattern 5, while clones no. 34, 41, and 43 showed patterns 6–8, respectively (Figure 1E). DNA sequences of four clones, i.e., 6, 10, 25, and 34, had complete coding regions, i.e., sequences of four FRs and three CDRs of both VH and VL domains with linker nucleotides coding for (G_4_S)_3_ in between the domains. Therefore, these clones were selected for further experiments.

### Cell Penetrable HuscFvs (transbodies) and Their Cell Penetrating Ability and Toxicity

**Figure 2:**
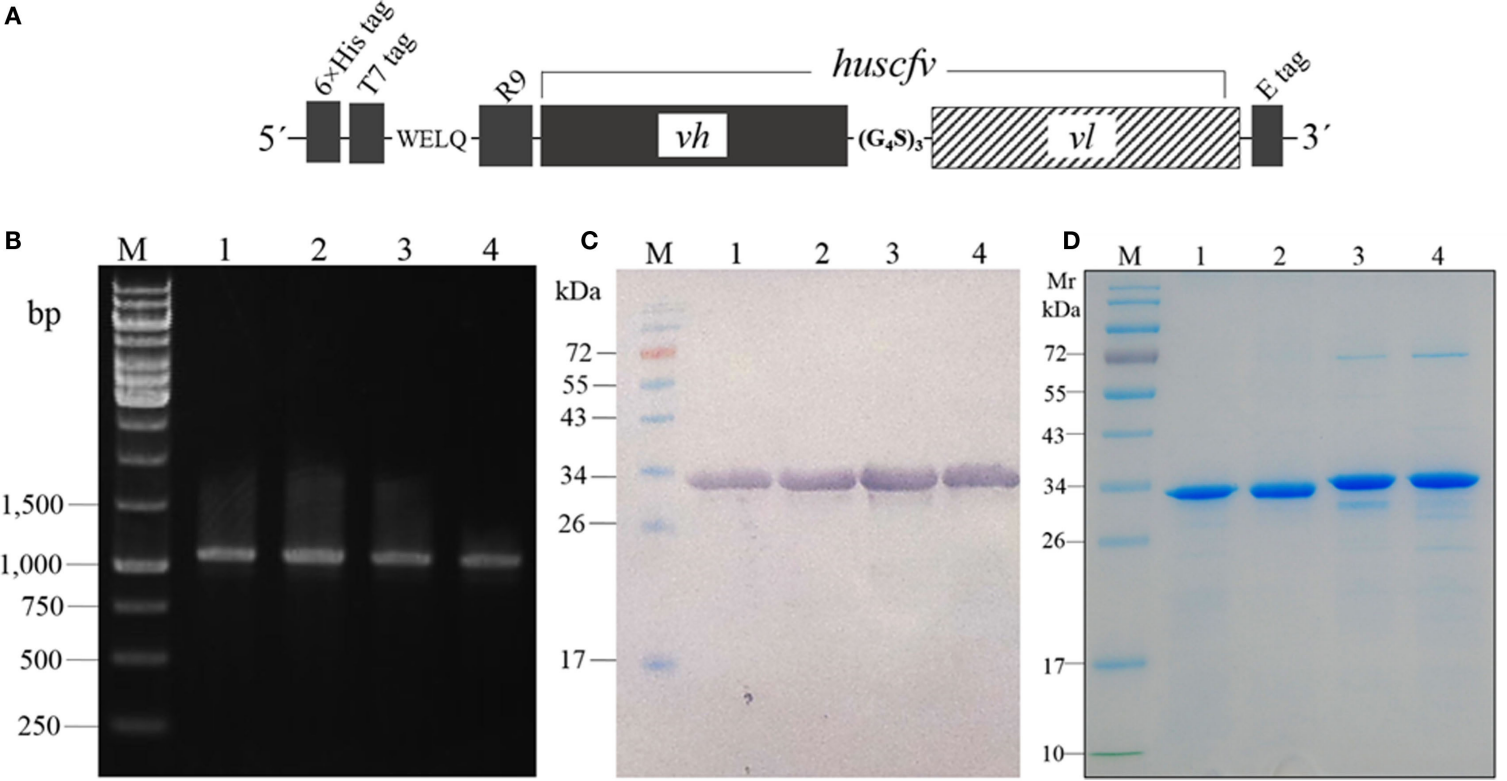
**Schematic diagram of the *R9-huscfv* DNA construct (A) which the DNA sequence coding for R9-HuScFv was flanked with 6× *His* and *T7* tags at the 5′ end and E-tag at the 3′ end; the *vh* and *vl* sequences of the *huscfv* were linked by nucleotides coding for (G_4_S)_3_**. PCR amplicons of *R9-huscfvs* contained in the pLATE52-*R9-huscfv* plasmid-transformed Rosetta™2 (DE3) *E. coli* clones **(B)**; the PCR product was ~1,140 bp; lane M, GeneRuler™ 1 kb DNA ladder; lanes 1–4, PCR amplicons of *R9-huscfv-6, -10, -25*, and *-34*, respectively; numbers at the left are DNA sizes in base pairs. Western blot patterns of homogenates of the four pLATE52-*R9-huscfv* plasmid-transformed Rosetta™2 (DE3) *E. coli* clones after growing under IPTG induction **(C)**; the homogenates were subjected to SDS-PAGE and probed with anti-6× His antibody; the R9-HuscFvs were ~34 kDa. Purified R9-HuscFv-6, -10, -25, and -34 from the respective *E. coli* inclusion bodies **(D)**.

The *huscfvs* of the clones 6, 10, 25, and 34 in the pCANTAB5E-*huscfv* recombinant phagemids were subcloned into the pLATE52 plasmids downstream of the R9 gene, and the recombinant plasmids were put into the Rosetta™2 (DE3) *E. coli*. Figure 2A shows schematic diagram of the R9-HuscFv DNA construct. Examples of the PCR products amplified from the pLATE52-*R9-huscfv-*transformed Rosetta™2 (DE3) *E. coli* sibling clones are shown in Figure 2B. The R9-HuscFvs with 6× His and T7 tags at the *N*-terminal and E-tag at the *C*-terminal were produced by growing the respective transformed *E. coli* clones under the 1 mM IPTG induction condition. All clones expressed the R9-HuscFv fusion antibodies at ~32–34 kDa (Figure 2C). Figure 2D shows SDS-PAGE-separated R9-HuscFvs after being purified and refolded. The purified transbodies retained the rNS3/4A binding activity in the indirect ELISA (data not shown).

**Figure 3:**
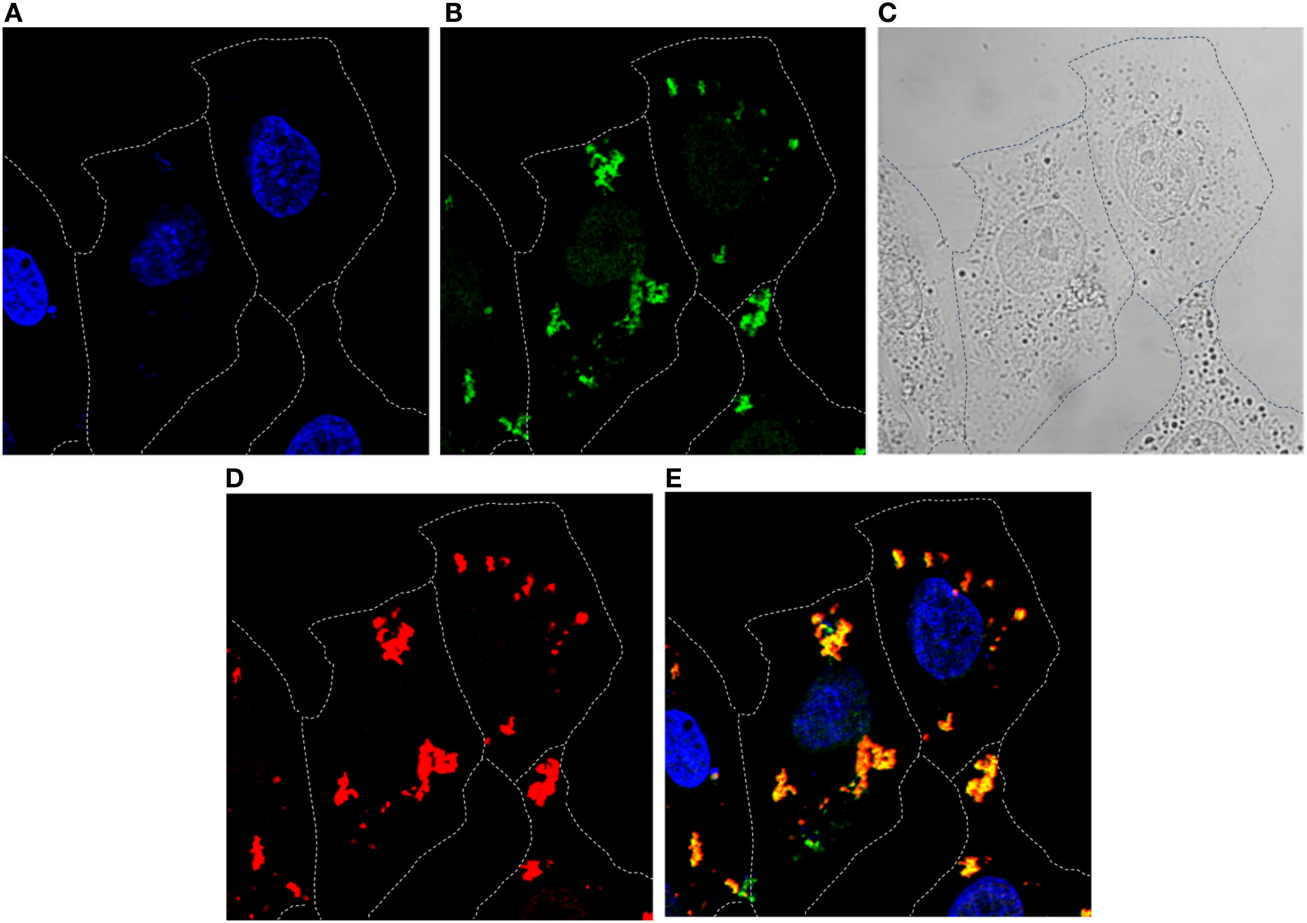
**Immunofluorescence staining and confocal microscopy for determining entry of R9-HuscFv10 (as representative) into HCV JFH-1 RNA-transfected Huh7 cells and the R9-HuscFv binding to the intracellular NS3/4A**. HCV JFH-1 RNA-transfected Huh7 cells were incubated with the R9-HuscFv then stained (see Materials and Methods). Cells stained with DAPI for localization of nuclei (blue) **(A)**. Intracellular R9-HuscFv probed with rabbit anti-6× His tag followed by goat anti-rabbit immunoglobulin-Alexa Flour^®^488 (Green) **(B)**. Bright field **(C)**. Intracellular NS3/4A probed with mouse anti-NS3/4A followed by donkey anti-mouse immunoglobulin-Alexa Flour^®^647 (red) **(D)**. Merged A, B, and D **(E)**. The R9-HuscFv readily entered the HCV-infected cells and colocalized with the intracellular NS3/4A (orange).

Ability of the R9-HuscFvs to enter the HCV JFH-1 RNA-transfected Huh7 cells and colocalized with the intracellular NS3/4A protein was determined by immunofluorescence assay and confocal microscopy. Representative result is shown in Figure 3. The intracellular R9-HuscFvs probed with rabbit anti-6× His tag followed by goat anti-rabbit immunoglobulin-Alexa Flour^®^488 gave green fluorescence, while the intracellular NS3/4A probed with mouse anti-NS3/4A followed by donkey anti-mouse immunoglobulin-Alexa Flour^®^647 appeared in red. The DAPI-stained nuclei are seen in blue. After merging, the R9-HuscFv and the NS3/4A were found to colocalize (orange).

**Figure 4:**
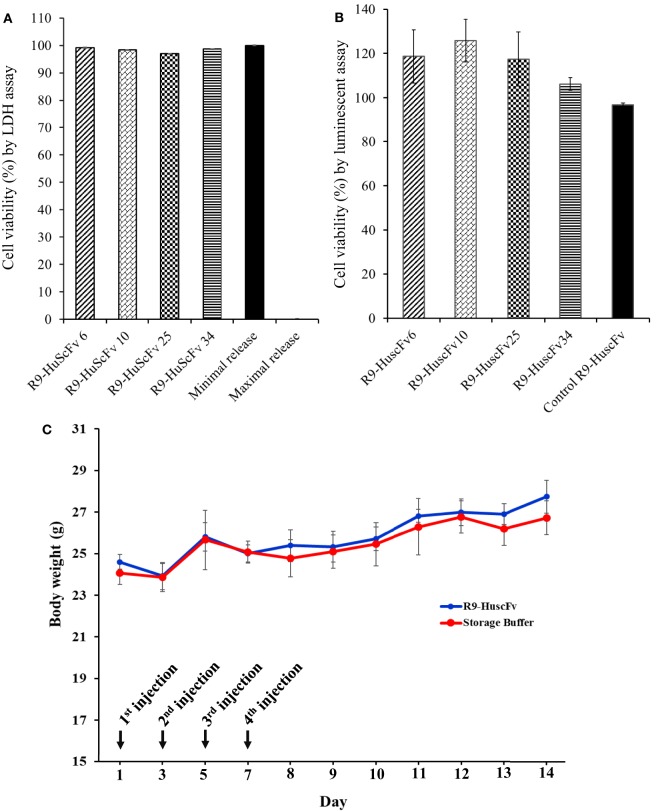
**Viability of the Huh7 cells after treating with R9-HuscFvs and controls as determined by LDH assay (A) and CellTiter-Glo^®^ Luminescent assay (B)**. For (A), Huh7 cells were incubated with 25 μg of R9-HuscFv-6, -10, -25, or -34. LDH in the cell culture medium was detected 24 h after adding the antibodies or controls, i.e., cells in medium alone (minimal/spontaneous LDH release) and cells added with 10% SDS (maximal LDH release). The calculated percent viability of the transbodies-treated cells was not different from the spontaneous (minimal) release. For (B), Huh7 cells were incubated with 25 μg of R9-HuscFvs or medium for 48 h and percent cell viability were determined using the Cell Titer-Glo^®^ Luminescent Cell Viability Assay kit. The R9-HuscFvs did not cause significant reduction of the cell viability compared with the cells cultured in the medium alone. Daily body weights of mice injected intraperitoneally **(C)** with R9-HuscFv10 (as representative; blue line) compared with control mice that were injected with buffer alone (red line); all mice gained some weights during 14 days of experiment and did not show any sign of morbidity. Some biochemical parameters of the mouse blood samples are shown in Table S1 in Supplementary Material.

Huh7 cells exposed to 25 μg of the respective R9-HuscFvs for 24 h did not release different LDH amounts from cells in medium alone (spontaneous release). The percent calculated viability of the transbody-treated cells was not different from the non-antibody-treated cells (Figure 4A). Likewise, percent cell viability after incubating with the R9-HuscFvs assessed using CellTiter-Glo luminescence assay were not different from the cells in culture medium alone (Figure 4B). Results indicate biocompatibility of the R9-HuscFvs with the mammalian cells.

None of the mice that received, individually, four doses of 25 μg/g body weight of R9-HuscFv10 (representative) and buffer intraperitoneally on every alternate day showed any sign of morbidity (fever, hair ruffle, or diarrhea). They had normal activity and appetite and gained some weight (Figure 4C). Values of some parameters of the mouse blood chemistry at day 7 post-last booster are shown in Table S1 in Supplementary Material.

### Transbody-mediated Inhibition of the HCV Replication

**Figure 5:**
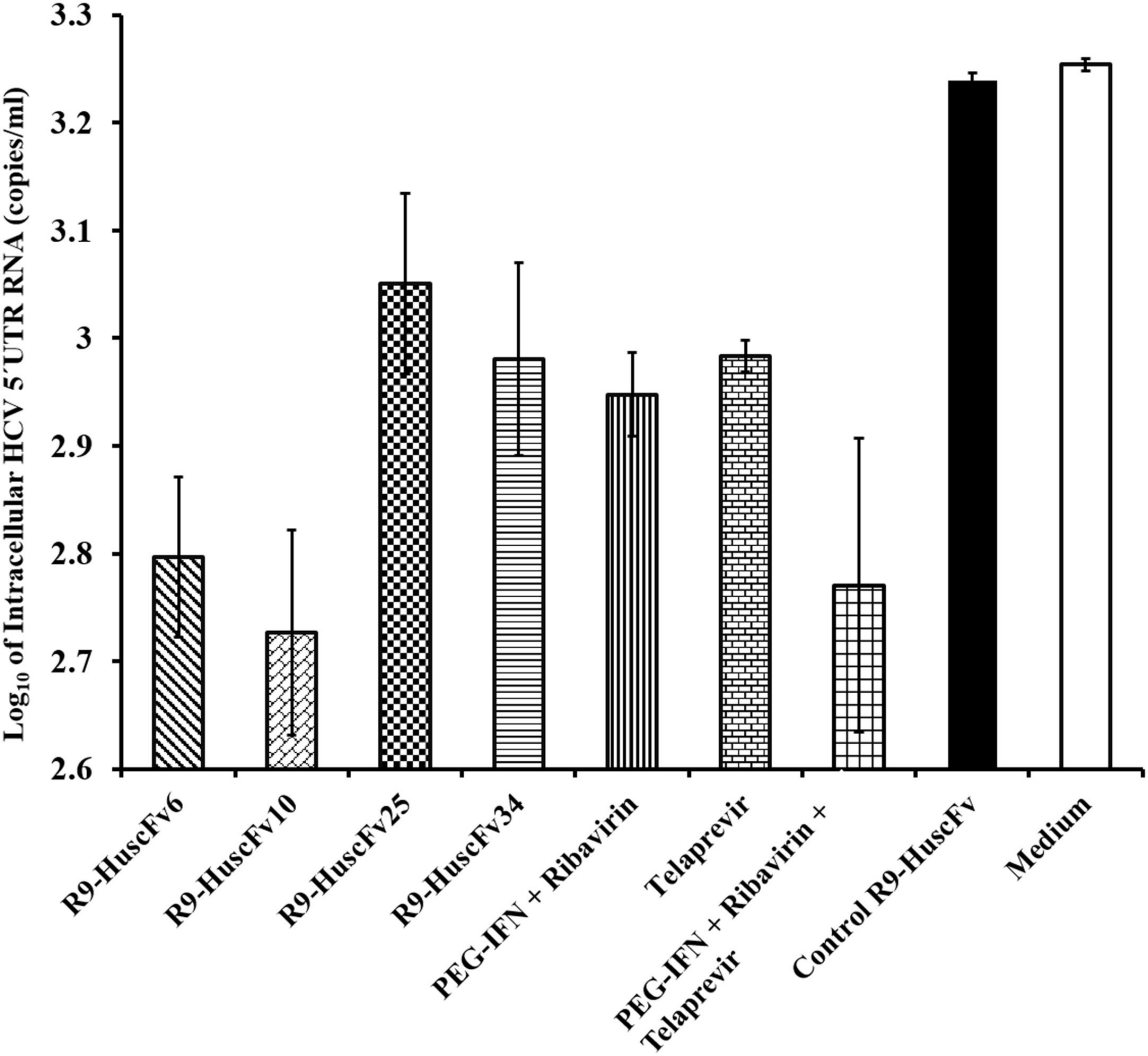
**Amounts of HCV 5′ UTR RNA in the JFH-1 RNA-transfected Huh7 cells after treating with the R9-HuscFv-6, -10, -25, and -34 in comparison with controls**. Table 1 shows *p* values for statistical comparison among different treatments.

The HCV 5′ UTR copy numbers recovered from the HCV-RNA-transfected Huh7 cells that had been exposed to the NS3/4A-bound-R9-HuscFv-6, -10, -25, and -34 are shown in Figure 5 in comparison with various controls (positive and negative inhibition controls). Among the R9-HuscFvs, the R9-HuscFv10 and R9-HuscFv6 were the most effective transbodies in reducing the amount of the intracellular HCV RNA. Efficacies of all inhibitors and controls in reducing the intracellular HCV RNA are statistically compared as shown in Table 1.

**Table 1:** **Statistical comparison of the results of the HCV 5′ UTR mRNA copy numbers recovered from the HCV-RNA-transfected Huh7 cells that had been exposed to the NS3/4A-bound R9-HuscFvs and various controls**.

Treatment	HuscFv					Positive inhibition control			Medium
	6	10	25	34	Control	PEG-IFN + ribavirin	Telaprevir	PEG-IFN + ribavirin + telaprevir	
**HuscFv**									
6	NS	*p* = 0.21	*p* < 0.05	*p* < 0.05	*p* < 0.05	*p* < 0.05	*p* < 0.05	*p* = 0.43	*p* < 0.05
10	*p* = 0.21	NS	*p* < 0.05	*p* < 0.05	*p* < 0.05	*p* < 0.05	*p* < 0.05	*p* = 0.64	*p* < 0.05
25	*p* < 0.05	*p* < 0.05	NS	*p* = 0.39	*p* < 0.05	*p* < 0.05	*p* = 0.34	*p* < 0.05	*p* < 0.05
34	*p* < 0.05	*p* < 0.05	*p* = 0.39	NS	*P* < 0.05	*p* = 0.23	*p* = 0.93	*p* < 0.05	*p* < 0.05
Control	*p* < 0.05	*p* < 0.05	*p* < 0.05	*p* < 0.05	NS	*p* < 0.05	*p* < 0.05	*p* < 0.05	*p* = 0.77
**Positive inhibition control**									
PEG-IFN + ribavirin	*p* < 0.05	*p* < 0.05	*p* < 0.05	*p* = 0.23	*P* < 0.05	NS	*p* = 0.27	*p* < 0.05	*p* < 0.05
Telaprevir	*p* < 0.05	*p* < 0.05	*p* = 0.34	*p* = 0.93	*p* < 0.05	*p* = 0.27	NS	*p* < 0.05	*p* < 0.05
PEG-IFN + ribavirin + telaprevir	*p* = 0.43	*p* = 0.64	*p* < 0.05	*p* < 0.05	*p* < 0.05	*p* < 0.05	*p* < 0.05	NS	*p* < 0.05
**Medium**	*p* < 0.05	*p* < 0.05	*p* < 0.05	*p* < 0.05	*p* = 0.77	*p* < 0.05	*p* < 0.05	*p* < 0.05	NS

*NS, not significantly different*.

**Figure 6:**
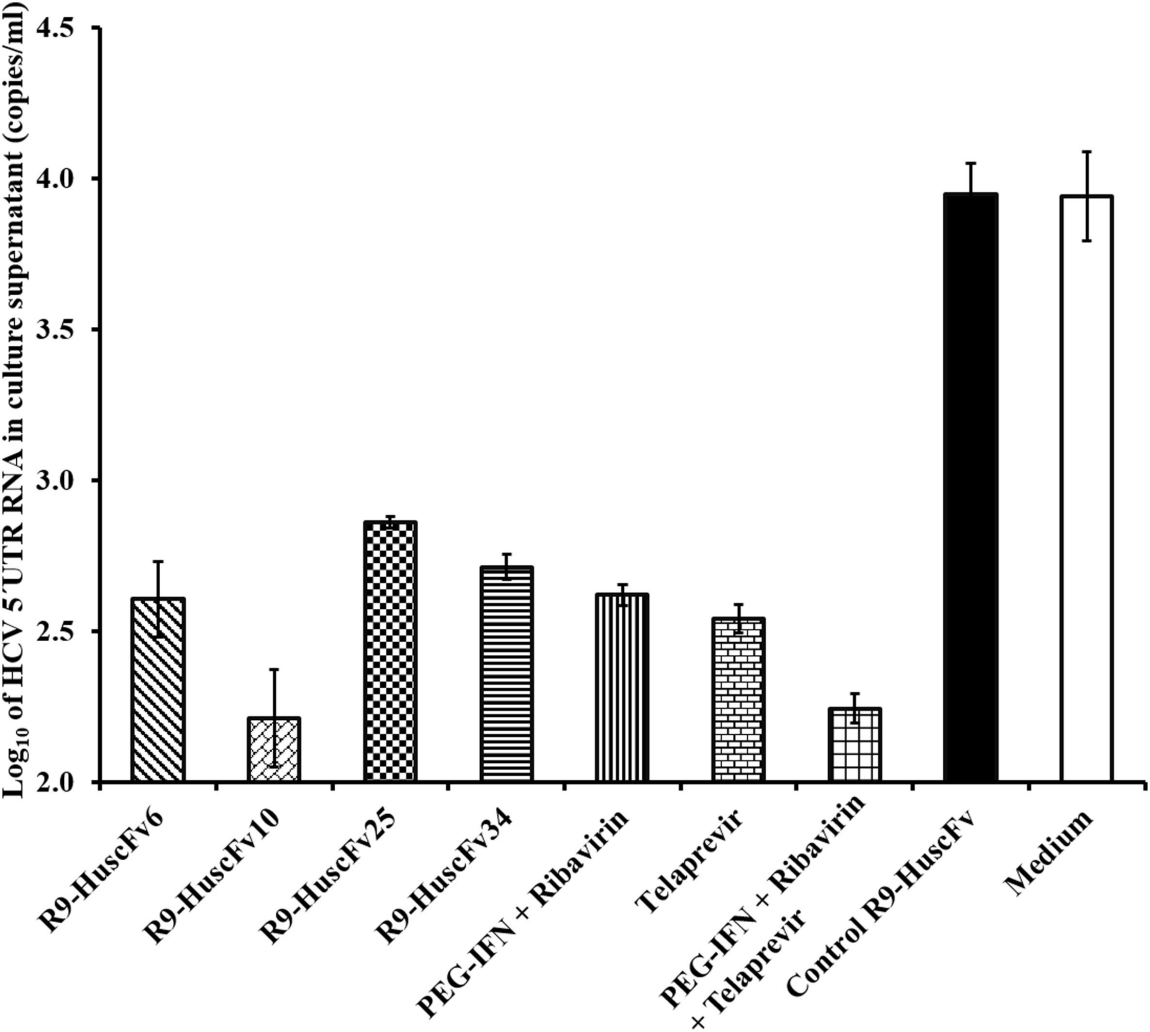
**Amounts of HCV 5′ UTR RNA in culture fluids after treating with the R9-HuscFv-6, -10, -25 and -34 in comparison with controls**. Table 2 shows *p* values for statistical comparison among different treatments.

Amounts of HCV 5′ UTR RNA in culture supernatants of all groups of the treated cells are shown in Figure 6. The most effective R9-HuscFvs in reducing the HCV RNA in the transfected cell culture supernatants were R9-HuscFv10 and R9-HuscFv6. Statistical differences of the effectiveness among groups are shown in Table 2.

**Table 2:** **Statistical comparison of the results of the HCV 5′ UTR copy numbers recovered from culture supernatants of the HCV-RNA-transfected Huh7 cells that had been treated with R9-HuscFv-6, -10, -25, and -34 and various controls**.

Treatment	HuscFv					Positive inhibition control			Medium
	6	10	25	34	Control	PEG-IFN + ribavirin	Telaprevir	PEG-IFN + ribavirin + telaprevir	
**HuscFv**									
6	NS	*p* < 0.05	*p* < 0.05	*p* = 0.23	*p* < 0.05	*p* = 0.88	*p* = 0.47	*p* < 0.05	*p* < 0.05
10	*p* < 0.05	NS	*p* < 0.05	*p* < 0.05	*p* < 0.05	*p* < 0.05	*p* < 0.05	*p* = 0.71	*p* < 0.05
25	*p* < 0.05	*p* < 0.05	NS	*p* < 0.05	*p* < 0.05	*p* = 0.24	*p* = 0.05	*p* < 0.05	*p* < 0.05
34	*p* = 0.23	*p* < 0.05	*p* < 0.05	NS	*P* < 0.05	*p* = 0.29	*p* = 0.17	*p* < 0.05	*p* < 0.05
Control	*p* < 0.05	*p* < 0.05	*p* < 0.05	*p* < 0.05	NS	*p* < 0.05	*p* < 0.05	*p* < 0.05	*p* = 0.94
**Positive inhibition control**									
PEG-IFN + ribavirin	*p* = 0.88	*p* < 0.05	*p* < 0.05	*p* = 0.29	*P* < 0.05	NS	*p* = 0.39	*p* < 0.05	*p* < 0.05
Telaprevir	*p* = 0.47	*p* < 0.05	*p* < 0.05	*p* < 0.05	*p* < 0.05	*p* = 0.39	NS	*p* < 0.05	*p* < 0.05
PEG-IFN + ribavirin + telaprevir	*p* < 0.05	*p* = 0.71	*p* < 0.05	*p* < 0.05	*p* < 0.05	*p* < 0.05	*p* < 0.05	NS	*p* < 0.05
**Medium**	*p* < 0.05	*p* < 0.05	*p* < 0.05	*p* < 0.05	*p* = 0.94	*p* < 0.05	*p* < 0.05	*p* < 0.05	NS

*NS, not significantly different*.

**Figure 7:**
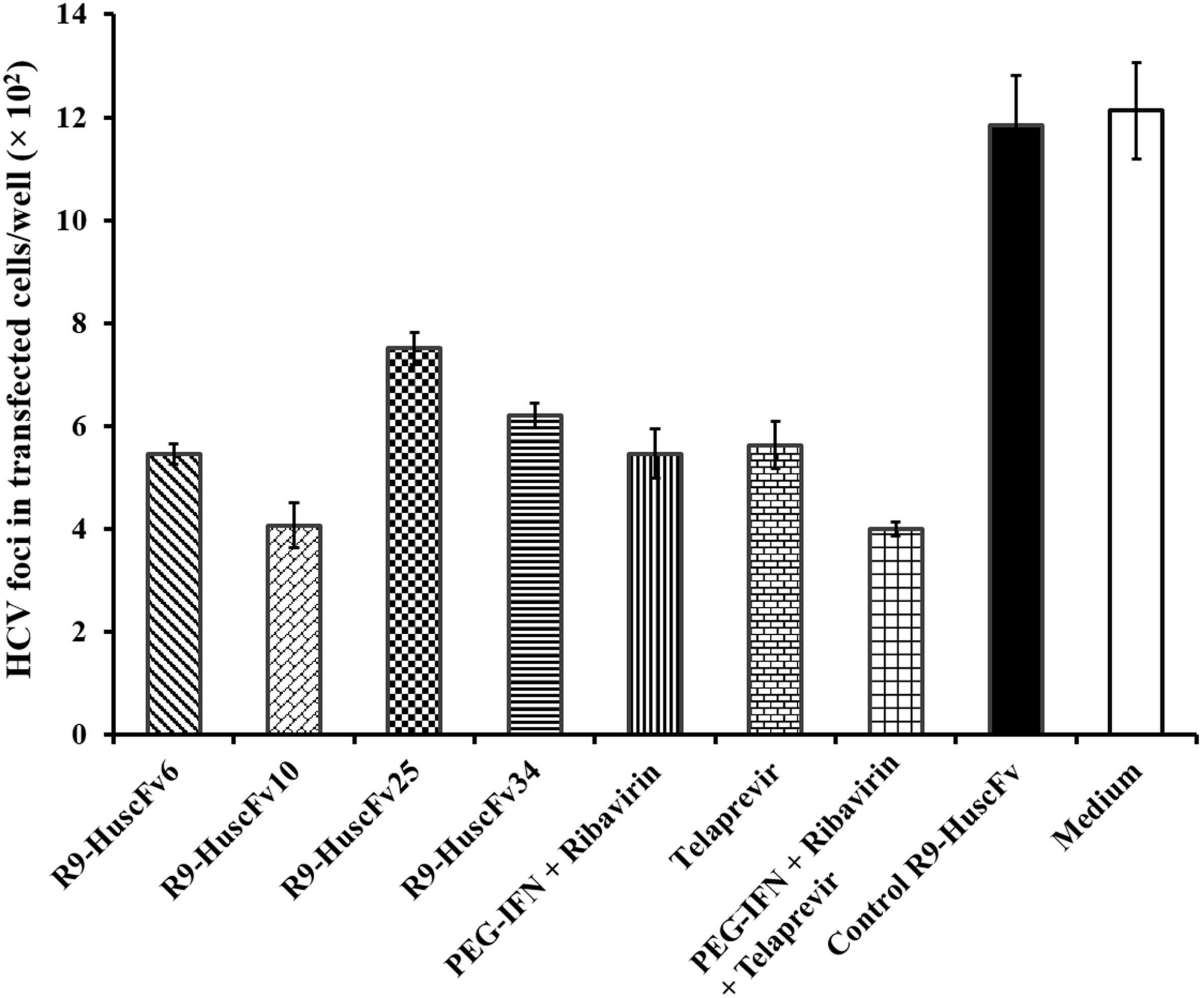
**Foci of HCV in the HCV-RNA-transfected Huh7 cells after exposure to the R9-HuscFv-6, -10, -25, and -34 in comparison with controls**. Table 3 shows *p* values for statistical comparison among different treatments.

Results of the assay for determining the HCV foci in transfected cells treated with R9-HuscFvs and controls are shown in Figure 7. Statistical comparison among all treatment groups are shown in Table 3. Figure S1 in Supplementary Material shows appearance of the HCV foci in cells of individual treatment groups after staining with anti-HCV core protein as the primary antibody. The effectiveness of the transbodies and the controls in reducing the numbers of HCV foci in the transfected cells were conformed to the results of the 5′ UTR RNA expression assay determined by the qRT-PCR. The R9-HuscFv10 was highly effective.

**Table 3:** **Statistical comparison of the results of the HCV foci assay among HCV-RNA-transfected Huh7 cells treated with R9-HuscFvs and various controls**.

Treatment	HuscFv					Positive inhibition control			Medium
	6	10	25	34	Control	PEG-IFN + ribavirin	Telaprevir	PEG-IFN + ribavirin + telaprevir	
**HuscFv**									
6	NS	*p* < 0.05	*p* < 0.05	*p* = 0.20	*p* < 0.05	*p* = 0.99	*p* = 0.75	*p* < 0.05	*p* < 0.05
10	*p* < 0.05	NS	*p* < 0.05	*p* < 0.05	*p* < 0.05	*p* < 0.05	*p* < 0.05	*p* = 0.90	*p* < 0.05
25	*p* < 0.05	*p* < 0.05	NS	*p* < 0.05	*p* < 0.05	*p* < 0.05	*p* < 0.05	*p* < 0.05	*p* < 0.05
34	*p* = 0.20	*p* < 0.05	*p* < 0.05	NS	*P* < 0.05	*p* = 0.20	*p* = 0.31	*p* < 0.05	*p* < 0.05
Control	*p* < 0.05	*p* < 0.05	*p* < 0.05	*p* < 0.05	NS	*p* < 0.05	*p* < 0.05	*p* < 0.05	*p* = 0.61
**Positive inhibition control**									
PEG-IFN + ribavirin	*p* = 0.99	*p* < 0.05	*p* < 0.05	*p* = 0.20	*P* < 0.05	NS	*p* = 0.77	*p* < 0.05	*p* < 0.05
Telaprevir	*p* = 0.75	*p* < 0.05	*p* < 0.05	*p* = 0.31	*p* < 0.05	*p* = 0.77	NS	*p* < 0.05	*p* < 0.05
PEG-IFN + ribavirin + telaprevir	*p* < 0.05	*p* = 0.90	*p* < 0.05	*p* < 0.05	*p* < 0.05	*p* < 0.05	*p* < 0.05	NS	*p* < 0.05
**Medium**	*p* < 0.05	*p* < 0.05	*p* < 0.05	*p* < 0.05	*p* = 0.61	*p* < 0.05	*p* < 0.05	*p* < 0.05	NS

*NS, not significantly different*.

### Response of the HCV-RNA-transfected Cells after Treatment with the Transbodies

Details of the fold change of mRNA expressions of the innate immune response genes, including *TRIF, TRAF3, IRF3, IL-28B*, and *IFN-*β, after various treatments in comparison with the non-transfected (normal) Huh7 cells cultured in the medium are shown in Table 4. The R9-HuscFv6 and R9-HuscFv10 restored expressions of all studied host innate immune response genes. The control transbody-exposed transfected cells and the transfected cells maintained in medium alone had lower expressions of the genes than the non-transfected (normal) cells. At the condition and time of the experiments, *TRAF3* and *IL-28*β mRNAs could not be rescued by the R9-HuscFv25 and R9-HuscFv34, respectively. All positive inhibitors, particularly the combined PEG-IFN-α + ribavirin + telaprevir effectively restored expressions of all of the studied genes.

**Table 4:** **Fold change of innate immune response genes in HCV-RNA-transfected human hepatic cells compared to non-transfected cells after treating with NS3/4A specific-R9-HuscFvs and various controls**.

Gene	Fold change of mRNAs of innate immune response genes in HCV-RNA-transfected cells treated with								
	R9-HuscFv				PEG-IFNα-2a + ribavirin	Telaprevir	PEG-IFNα-2a + ribavirin + telaprevir	Control R9-HuScFv	Medium
	6	10	25	34					
*TRIF*	45.89↑	49.52↑	3.23↑	1.25↑	28.64↑	10.78↑	150.12↑	1.18↓	1.08↓
*TRAF3*	32.67↑	83.87↑	1.75↓	1.97↑	15.14↑	11.96↑	64.89↑	1.23↓	1.82↓
*IRF3*	22.78↑	32.90↑	3.71↑	3.05↑	8.69↑	11.96↑	26.09↑	5.55↓	1.11↓
*IL-28B*	15.67↑	10.41↑	1.89↑	3.57↓	22.16↑	6.11↑	34.06↑	3.70↓	2.00↓
*IFN*β*1*	16.45↑	51.98↑	6.36↑	4.66↑	53.08↑	18.90↑	125.37↑	1.64↓	1.32↓

*↑, fold increase; ↓, fold decrease*.

### Homology Modeling and Intermolecular Docking for Predicting the Presumptive Residues of the HCV NS3/4A Interacted with the HuscFvs

The top 10 treading templates used to model the HuscFv6, HuscFv10, HuscFv25, and HuscFv34 by I-TASSER and the top 10 identified structural analogs of the HuscFvs in the PDB are shown in Tables S2A,B–S5A,B in Supplementary Material, respectively. Table S6 in Supplementary Material gives details of the qualities of the I-TASSER-predicted HuscFv models used in this study. The ClusPro lowest local energies of interactions between the NS3/4A 3D structure and the modeled HuscFv6, HuscFv10, HuscFv25, and HuscFv34 were −356, −428, −290, and −293 kcal/mol, respectively. Details of intermolecular docking, including the interactive residues of the NS3/4A/HCV polyprotein and motives and amino acids and domains of the HuscFvs as well as the interactive bonds between the two parties, are shown in Table 5 and Figures S2–S5 in Supplementary Material. Besides the molecular bonds in Table 5, the amino acid residues of the NS3/4A within 5 Å thresholds of van der Waals radii of HuscFvs are shown separately in Table 6.

**Table 5:** **Presumptive residues and regions of the NS3/4A and polyprotein of HCV bound by specific HuscFvs**.

HCV protein			HuscFv		Intermolecular bond
NS3 residue	Polyprotein residue	Motif (-ves)	Amino acid	Domain	
**A. HuscFv6**					
R117	R1149	β-B2	F52	VH-CDR2	Cation-π
			Y102	VH-CDR3	H-bond
R118	R1150	β-B2	Y102	VH-CDR3	CH-π
R119	R1151	Between β-B2 and β-C2	D33	VH-CDR1	Salt-bridge
			S57	VH-CDR2	H-bond
D121	D1153	Between β-B2 and β-C2	Y166	VL-CDR1	H-bond
S122	S1154	Between β-B2 and β-C2	S227	VL-CDR3	H-bond
			F228	VL-CDR3	H-bond
R123	R1155	Between β-B2 and β-C2	E59	VH-CDR2	H-bond
			E62	VH-CDR2	Salt-bridge
			F228	VH-CDR3	NH-π
K165	K1197	β-F2	E55	VH-CDR2	Salt-bridge
			S57	VH-CDR2	H-bond
P171	P1203	β-F2	Y226	VH-CDR3	CH-π
E173	E1205	α-helix c	Y226	VL-CDR3	H-bond
**B. HuscFv10**					
T40	T1071	Between β-B1 and β-C1	E55	VH-CDR2	H-bond
Y56	Y1088	α-helix a	Y227	LCDR3	π–π stacking
H57	H1089	α-helix a (catalytic triad)	Y165	LCDR1	H-bond
D79	D1111	Between β-E1 and β-F1	S162	LCDR1	Water bridge
			R164	LCDR1	H-bond
			S225	LCDR3	H-bond
D81	D1113	Between β-E1 and β-F1 (catalytic triad)	Y165	LCDR1	H-bond
K136	K1168	Between α-helix b and β-D2 (oxyanion loop)	S31	VH-CDR3	H-bond
			Y101	VH-CDR3	Cation-π
G137	G1169	Between α-helix b and β-D2 (oxyanion loop)	Y102	VL-CDR1	H-bond
R155	R1187	β-E2	Y165	VH-CDR	H-bond
			D183	VL-CDR2	Salt-bridge
A157	A1189	β-E2	Y105	VH-CDR3	H-bond
NS4A K20	K1677	Close to the first residue of NS4A cofactor peptide that binds to NS3 for enhancing the protease activity	T58	VH-CDR2	H-bond
			E59	VH-CDR2	Salt-bridge
**C. HuscFv25**					
D103	D1135	β-A2	R31	VH-CDR1	Salt-bridge
R117	R1149	β-B2	F101	VH-CDR3	NH-π
R119	R1151	Between β-B2 and β-C2	R50	VH-CDR2	Water bridge
			E99	VH-CDR3	Salt-bridge
			F101	VH-CDR3	H-bond
			Y168	VL-CDR2	H-bond
			Y232	VL-CDR3	H-bond
D121	D1153	Between β-B2 and β-C2	D164	VL-CDR1	H-bond
S122	S1154	Between β-B2 and β-C2	D164	VL-CDR1	H-bond
R123	R1155	Between β-B2 and β-C2	S163	VL-CDR1	H-bond
			D164	VL-CDR1	H-bond
S125	S1157	β-C2	R50	VH-CDR2	Water bridge
L127	L1159	β-C2	W33	VH-CDR1	CH-π
			T52	VL-CDR2	H-bond
K165	K1197	β-F2	D57	VL-CDR2	Water bridge
			D59	VL-CDR2	Salt-bridge
			Y60	VL-CDR2	H-bond
			W230	VL-CDR3	CH-π
**D. HuscFv34**					
S102	S1134	Between β-F1 and β-A2	D57	VH-CDR1	H-bond
D103	D1135	β-A2	D57	VH-CDR2	H-bond
R117	R1149	β-B2	T58	VH-CDR2	H-bond
			D59	VH-CDR2	H-bond
R118	R1150	β-B2	W50	VH-CDR2	CH-π
R119	R1151	Between β-B2 and β-C2	D59	VH-CDR2	Salt-bridge
D121	D1153	Between β-B2 and β-C2	S101	VH-CDR3	H-bond
			D182	VH-CDR2	Water bridge
			R223	VL-CDR3	H-bond
S122	S1154	Between β-B2 and β-C2	R233	VL-CDR3	H-bond
P146	P1178	Between β-D2 and β-E2 (inside zinc binding site)	Y54	VH-CDR2	CH-π
T147	T1179	Between β-D2 and β-E2 (inside zinc binding site)	Y54	VH-CDR2	CH-π
E173	E1205	α-helix c	K103	VH-CDR3	H-bond

**Table 6:** **Amino acid residues of NS3 within 5 Å threshold of van der Waals radii of HuscFvs in Table 2**.

HCV protein	
NS3 residue(s)	NS3 motif (-ves)
**A. HuscFv6**	
D79	Between β-E1 and β-F1
G100, S101, S102	Near the zinc-binding site (C99)
D103	β-A2
G120	Between β-B2 and β-C2
G124, S125, L126, L127	β-C2
R155, V158	β-E2
A166, D168, D169	β-F2
N174, E176, T177, R178	α-helix c
R155, V158	β-E2
A166, D168, D169	β-F2
N174, E176, T177, R178	α-helix c
**B. HuscFv10**	
L13	Between β-A0 and α-helix 0
I17	α-helix 0
A39, Q41	Between β-A1 and β-B1
T42, F43	β-B1
V55	β-C1
G58, A59	α-helix a (near the catalytic site, H57)
G60, T61, R62	Between α-helix a and β-E1
V78, Q80	Between β-E1 and β-F1 (near catalytic site, D81)
R123	Between β-B2 and β-C2 (S6 pocket)
I132, S133, L135	α-helix b; L135 is at the S1 pocket
S138	Oxyanion loop
S139	Catalytic triad
F154, A156, V158, C159	β-E2 (F154 is at the S1 pocket)
T160, R161	Between β-E2 and β-F2
A164, D168	β-F2
**C. HuscFv25**	
S101	Near the zinc binding site (C99)
Y105	β-A2
P115, R118	β-B2
G120	Between β-B2 and β-C2
G124, L126, S128, P129	β-C2
P146	Near the zinc binding site (C145, H149)
V158	β-E2
T160, R161, V163	Between β-E2 and β-F2
A166	β-F2
**D. HuscFv34**	
D79, Q80	Between β-E1 and β-F1 (near catalytic site, D81)
C97	Zinc-binding site
T98	Between zinc-binding site (C97, C99)
C99	Zinc-binding site
G100, S101	Near zinc-binding site
G120, R123	Between β-B2 and β-C2 (R123 is at the S6 pocket)
S125	β-C2
C145	Zinc-binding site
P171	β-F2
V172, N174, T177	α-helix c
R18	Near α-helix c

The most effective HuscFv10 in inhibiting the HCV replication was found to form interface contact with many critical residues of the NS3/4A important for protease activity including H57 and D81 of the catalytic triad, Y56 and D79 near to the catalytic site, K136 and G137 of the oxyanion loop, and K20 of NS4A cofactor which is adjacent to the NS4A residues 21–34 that usually interacts with the NS3 for enhancing the protease activity (Table 5B and Figure S2 in Supplementary Material). Table 6B shows the van de Waals forces that may contribute also to the HuscFv10-target association.

The HuscFv6, which was also highly effective in HCV replication inhibition, did not interact with any residues of the catalytic triad (H57, D81, and S139), oxyanion loop (135LKGSS139), or residues C97, C99, C145, and H149 of the zinc binding site. Instead, center of target binding of this HuscFv6 was located at the basic triad, i.e., 117RRR119, and R123 of the NS3/4A β–B2 and β-C2 motives (Tables 5A and 6A and Figure S3 in Supplementary Material).

The HuscFv25, similar to the HuscFv6, interacted with the basic residues: R117, R119, and R123, although at the higher local energy than the HuscFv6 (Tables 5C and 6C and Figure S4 in Supplementary Material).

The HuscFv34 not only interacted with the 117RRR119 but also with the P146 and T/S147 in the zinc binding site of the protease (Tables 5D and 6D and Figure S5 in Supplementary Material).

## Discussion

There is a need of an improved regimen/remedy for treatment of HCV infection. A novel DAA that we would like to offer for future development is the engineered cell-penetrating human/humanized small antibody fragments that interfere with enzymes of the virus. The NS3/4A serine protease is pivotal for releasing mature NS4A, NS4B, NS5A, and NS5B from polyprotein (42) for the HCV replication, assembly, and egress. It also suppresses the host innate antiviral response.

During the early phase of infection, molecular signatures of intracellular viruses usually alarm the host pathogen recognition receptors (PRRs), including endosomal Toll-like receptors (TLRs), and cytosolic retinoic acid-inducible gene 1/helicard (RIG-1/Mda5) to activate various adaptor proteins, i.e., TRIF (Toll-IL-1 receptor domain-containing adaptor inducing IFN-β), Cardif/MAVS, and TRAFs (tumor necrosis factor-associated factors). These factors consequently activate kinases (TBK1 and IKKs) leading to phosphorylation and dimerization of interferon regulatory factor 3/7 (IRF3/7) transcription factor for production of innate interferons, including type-I IFN-β and type-III IFN-λ (IL-28/29) (43–45). The IFNs mediate autocrine and paracrine stimulation of the host cells through JAK/STAT signaling pathways leading to production of various antiviral factors, i.e., 2′, 5′ OAS, PKR, MxA, and ISGs, as well as MHC class I molecules for cytotoxic lymphocyte activation (45). NS3/4A protein of the infecting HCV cleaves TRIF and Cardif/MAVS, binds to TBK1, and inhibits activation of IRF3 (46–48). Moreover, the protein has been implicated in malignant transformation (49–51). The NS3 *N*-terminal is known to interact with p53 (a tumor suppressor) and inhibited apoptosis of NIH3T3 cells (51, 52). As such, interference with the HCV NS3/4A functions should lead to inhibition of the viral replication as well as restoration of the virally suppressed host innate immune response and might as well reduce the hepatocellular carcinoma incidence among the chronically infected subjects.

In this study, bacterially derived engineered human single chain antibodies that bound to active HCV NS3/4A protein were generated. Because the antibody target is located intracellularly, the antibodies were made into cell penetrable molecules by linking their coding sequences to nonaarginine (R9) nucleotides using a ligase-independent cloning (LIC) method. The R9-HuscFvs were readily purified from the IBs of four *E. coli* clones (nos. 6, 10, 25, and 34) carrying the *R9-huscfv* plasmids and successfully refolded using the protocol adapted from (53) and instruction manual of the Protein Refolding Kit of Novagen. The R9-HuscFvs retained their binding to the rNS3/4A as the parental HuscFvs (data not shown) indicating the native refolding of the antibodies. They readily entered the human hepatic cells (Huh7) and bound to the intracellular target. They did not cause significant LDH leakage from the cocultured human hepatic cells compared with the cells in the medium alone indicating compatibility of the antibody preparations with the mammalian cells at the dose that was tested. Safety of the transbodies was also demonstrated in mice after injecting them intraperitoneally (followed the Thai Pharmacopia) at the dose that was relatively high (25 μg/g body weight) for passive immunization in humans (37).

There were significant reductions of the HCV 5′UTR RNA in the Huh7 cells transfected with JFH-RNA and in the cell culture supernatants after treating with the R9-HuscFvs (albeit different degrees of effectiveness among the transbodies from different *E. coli* clones). The results of foci assay were conformed to the RNA quantification. The most effective transbodies were R9-HuscFv10 and R9-HuscFv6. These transbodies could readily restore expressions of all of the studied innate immune response genes of the HCV-RNA-transfected cells. Taken all together, the results indicate that the transbodies-mediated viral replication inhibition and host innate immune response restoration *via* the NS3/4A protease interference.

Presumptive residues of the NS3/4A interacted with the HuscFvs were determined by means of computerized homology modeling and intermolecular docking. The lowest local energy of interaction between the HuscFv10 (which was most effective in inhibiting the HCV replication and in restoration of the host innate gene expressions) and the NS3/4A was −428 kcal/mol, indicating relatively higher affinity on the target binding of this antibody than the other three antibodies. The predictive residues of the NS3/4A protease interacted by the HuscFv10 included several residues critical for the protease activity, i.e., H57 and D81 of the catalytic triads and K136 and G137 of the oxyanion loop (54, 55). Binding of the antibody to the H57 and D81 should inhibit deprotonation of the S139 and, thus, failure of the S139 to mediate a nucleophilic attack of the carbonyl carbon of the scissile bond to form a tetrahedral intermediate containing an oxyanion; therefore, the proteolytic cleavage should not be complete, and, hence, no release of the N- and C-peptide products from the polyprotein (56). The HuscFv10 might as well disturb the function of the oxyanion loop by docking on the K136 and G137. Moreover, binding of the antibody to the NS4A K20 might as well interfere with the non-covalent interaction between the cofactor (residues 21–34 of NS4A) to the NS3; thus, impaired the cofactor enhancement of the protease activity. Besides, van de Waals force formed by HuscFv10 and several residues of NS3/4A molecule (Table 6) might have additional negative impact on the protease activity. Possibly by causing steric hindrance of the oxyanion hole *via* the S138, the S1 pocket through interactive atoms at the L135, F154 and nearby residues, i.e., A156, V158, and C159 (54, 55), and the S6 pocket by means of R123 which is one of the positively charged residues of the S6 that usually interact with the P6 and P5 residues (56, 57).

The HuscFv6 was as effective as the HuscFv10 in inhibiting the HCV replication. The antibody readily restored the host innate immune response genes from the HCV suppression as did the R9-HuscFv10. From computerized simulation, the lowest local binding energy of this antibody to the NS3/4A was as low as −356 kcal/mol. However, computerized simulation indicated that the HuscFv6 did not dock on any residues of the catalytic triad, oxyanion hole, or zinc biding site. Instead, the center of interaction of this antibody with the target was at the basic patch of the NS3 protein formed by R117, R118, and R119. Although the role of this cationic patch has not been elucidated, it is likely that residues in this region might form electrostatic interaction with certain negatively charged surface, such as viral RNA. This interaction might mediate pivotal, yet unraveled, activity of the HCV NS3. Binding of the R9-HuscFv6 to these residues might interfere with the activity. Besides, the positively charged residues R123 and K165 of the NS3/4A S6 pocket are believed to interact with acidic residues at the P6, and sometimes the P5 also (57, 58); blocking of the R123 and K165 by CDR1 and CDR2 of the R9-HuscFv6 VL domain (Table 2) might interfere with the substrate binding of the protease. The HuscFv6 was predicted to form interactive atoms of van de Waals radii with multiple residues of the target (Table 3) that might also interfere with the NS3/4A activity by steric hindrance.

The HuscFv25 was the least effective transbody among the four in inhibiting the HCV replication. Computerized intermolecular docking revealed that the antibody also interacted with the protease basic patch (R117, R118, and R119) and S6 pocket residues (R123 and K165) similar to the HuscFv6. However, the local energy that required for binding of this antibody to the target (−290 kcal/mol) was higher than that of the HuscFv6, which might explain the lower effectiveness of the HuscFv25.

The HuscFv34 interacted with residues of the zinc binding site (C97, C99, C145, P146, and T147), which might cause improper folding of the NS3 and, therefore, deficient in the protease activity (59, 60). The antibody also docked on the NS3 basic patch and, thus, might interfere with the important, but still unknown, activity of the NS3 protein.

In conclusion, bacterially derived cell-penetrating human single chain antibodies specific to NS3/4A protein of HCV produced in this study inhibited replication of the heteologous HCV (genotype 2a of the pJFH-1) in human hepatic cells and restored expressions of the virally suppressed host innate immune response genes. They were not toxic to human cells and mice. The transbodies have potential for developing and testing further for anti-HCV activity, either alone, in combination with the approved HCV therapeutics, or in a mixture with their cognate specific to other HCV proteins.

## Ethics Statement

Animal experiments received approval from the Siriraj Bio-safety Risk Management Taskforce (SI 2014-004), Faculty of Medicine Siriraj Hospital, Mahidol University, Bangkok 10700, Thailand. The consent procedure used for human participants is not applicable.

## Author Contributions

SJ, WS, JT, and KT-i did experiments and prepared figures; RW, supervised SJ on protein purification; WC conceived the project, designed experiments, analyzed data, and wrote the manuscript; PS helped WC on experimental design.

## Conflict of Interest Statement

The authors declare that the research was conducted in the absence of any commercial or financial relationships that could be construed as a potential conflict of interest.
