# Supplementary material for: Human Transbodies to HCV NS3/4A Protease Inhibit Viral Replication and Restore Host Innate Immunity
Source: Front Immunol. 2016 Aug 26;7:318. doi: 10.3389/fimmu.2016.00318 (PMC4999588; doi:10.3389/fimmu.2016.00318)
Supplement: Supplementary file 1 [file Table_1.DOCX]

**Supplementary Table 1.** Some clinical chemistry values of male BALB/c mice at day 7 after the last dose of R9-HuscFv10 or buffer.

| **Clinical chemistry values** | **R9-HuscFv injected mice** | **Buffer injected mice** | ***P* value** |
| --- | --- | --- | --- |
| Alkaline phosphatase (U/L) | 151.60 ± 25.01 | 201.25 ± 11.87 | *p* = 49.65 |
| Alanine aminotransferase (U/L) | 118.00 ± 52.42 | 86.25 ± 26.09 | *p* = 31.75 |
| Aspartate aminotransferase (U/L) | 168.80 ± 34.64 | 149.50 ± 70.07 | *p=* 19.30 |
| Total birirubin (mg/dL) | 1.02 ± 0.36 | 0.88 ± 0.22 | *p=* 0.145 |

U/L, units/liter; *p* < 0.05 was different statistically
