# Supplementary material for: Human Transbodies to HCV NS3/4A Protease Inhibit Viral Replication and Restore Host Innate Immunity
Source: Front Immunol. 2016 Aug 26;7:318. doi: 10.3389/fimmu.2016.00318 (PMC4999588; doi:10.3389/fimmu.2016.00318)
Supplement: Supplementary file 2 [file Table_2.DOCX]

**Supplementary Table 2A.** Top 10 threading templates used to model HuscFv6 by I-TASSER

| **Rank** | **PDB Hit** | **Iden1** | **Iden2** | **Cov** | **Norm. Z-score** |
| --- | --- | --- | --- | --- | --- |
| 1 | 3umtA | 0.67 | 0.64 | 0.94 | 3.48 |
| 2 | 3umtA | 0.67 | 0.64 | 0.94 | 4.67 |
| 3 | 4h0gA | 0.63 | 0.62 | 0.98 | 3.57 |
| 4 | 3mutA | 0.67 | 0.64 | 0.94 | 0.78 |
| 5 | 4hjjL | 0.41 | 0.39 | 0.91 | 0.83 |
| 6 | 3mutA | 0.67 | 0.64 | 0.94 | 3.34 |
| 7 | 4hjjL | 0.41 | 0.39 | 0.90 | 1.28 |
| 8 | 3mutA | 0.67 | 0.64 | 0.94 | 6.29 |
| 9 | 3mutA | 0.67 | 0.64 | 0.94 | 2.51 |
| 10 | 2gkiA | 0.68 | 0.64 | 0.94 | 5.03 |

**Rank,** Rank of templates represents the top ten threading templates used by I-TASSER.

**Ident1,** The percentage sequence identity of the templates in the threading aligned region with the query sequence

**Ident2**: The percentage sequence identity of the whole template chains with query sequence

**Cov**, The coverage of the threading alignment which is equal to the number of aligned residues divided by the length of query protein

**Norm. Z-score,** the normalized Z-score of the threading alignments. Alignment with a Normalized Z-score >1 mean a good alignment

The top 10 alignments reported above (in order of their ranks) are from the following threading programs:1) MUSTER, 2) FFAS-3D, 3)SPARKS-X, 4) HHSEARCH2, 5) HHSEARCH I, 6) Neff-PPAS , 7) HHSEARCH, 8) pGenTHREADER, 9) wdPPAS, and 10) cdPPAS

**Supplementary Table 2B.** Top 10 identified structural analogs of target HuscFv6 in PDB

| **Rank** | **PDB Hit** | **TM-score** | **RMDS^a^** | **IDEN^a^** | **Cov** |
| --- | --- | --- | --- | --- | --- |
| 1 | 4h0gA | 0.937 | 1.13 | 0.627 | 0.963 |
| 2 | 3umtA | 0.935 | 0.52 | 0.674 | 0.942 |
| 3 | 3juyB | 0.921 | 1.32 | 0.554 | 0.959 |
| 4 | 2ghwB | 0.905 | 1.29 | 0.559 | 0.942 |
| 5 | 1qokA | 0.903 | 1.21 | 0.591 | 0.934 |
| 6 | 3gm0A | 0.900 | 2.00 | 0.517 | 0.971 |
| 7 | 4f9pD | 0.900 | 1.14 | 0.643 | 0.929 |
| 8 | 3uzvB | 0.890 | 1.26 | 0.576 | 0.929 |
| 9 | 4buhA | 0.890 | 1.33 | 0.527 | 0.929 |
| 10 | 1dzbA | 0.884 | 1.36 | 0.653 | 0.921 |

**Rank,** rank of proteins based on TM-score of the structural alignment between the query structure and known structures in the PDB library

**RMSD^a^,** the RMSD between residues that are structurally aligned by TM-align

**IDEN^a^,** the percentage sequence identity in the structurally aligned region

**Cov,** the coverage of the alignment by TM-align that is equal to the number of structurally aligned residues divided by length of the query protein

**Supplementary Table 3A.** Top 10 threading templates used to model HuscFv10 by I-TASSER

| **Rank** | **PDB Hit** | **Iden1** | **Iden2** | **Cov** | **Norm. Z-score** |
| --- | --- | --- | --- | --- | --- |
| 1 | 3gm0A | 0.57 | 0.58 | 0.97 | 3.54 |
| 2 | 4h0gA | 0.61 | 0.60 | 0.98 | 4.58 |
| 3 | 4h0gA | 0.61 | 0.60 | 0.98 | 3.60 |
| 4 | 3gkzA | 0.59 | 0.56 | 0.95 | 0.78 |
| 5 | 4hjjL | 0.40 | 0.39 | 0.91 | 0.83 |
| 6 | 3gm0A | 0.57 | 0.58 | 0.97 | 3.32 |
| 7 | 4hjjL | 0.40 | 0.39 | 0.90 | 1.27 |
| 8 | 4h0hB | 0.60 | 0.60 | 0.97 | 6.10 |
| 9 | 3umtA | 0.63 | 0.59 | 0.94 | 2.58 |
| 10 | 2gkiA | 0.64 | 0.61 | 0.94 | 5.40 |

**Supplementary Table 3B.**  Top 10 identified structural analogs of target HuscFv10 in PDB

| **Rank** | **PDB Hit** | **TM-score** | **RMDS^a^** | **IDEN^a^** | **Cov** |
| --- | --- | --- | --- | --- | --- |
| 1 | 4h0gA | 0.938 | 1.07 | 0.613 | 0.959 |
| 2 | 3gm0A | 0.930 | 1.04 | 0.581 | 0.971 |
| 3 | 3juyB | 0.918 | 1.24 | 0.578 | 0.954 |
| 4 | 3umtA | 0.916 | 0.94 | 0.633 | 0.938 |
| 5 | 1qokA | 0.914 | 0.97 | 0.702 | 0.934 |
| 6 | 2ghwB | 0.907 | 1.20 | 0.559 | 0.942 |
| 7 | 4buhA | 0.893 | 1.31 | 0.549 | 0.929 |
| 8 | 4f9pD | 0.893 | 1.18 | 0.628 | 0.925 |
| 9 | 3uzqA | 0.891 | 1.57 | 0.575 | 0.946 |
| 10 | 1dzbA | 0.889 | 1.17 | 0.652 | 0.917 |

**Supplementary Table 4A.** Top 10 threading templates used to model HuscFv25 by I-TASSER

| **Rank** | **PDB Hit** | **Iden1** | **Iden2** | **Cov** | **Norm. Z-score** |
| --- | --- | --- | --- | --- | --- |
| 1 | 2ghwB | 0.66 | 0.63 | 0.93 | 3.46 |
| 2 | 4h0gA | 0.61 | 0.60 | 0.98 | 4.39 |
| 3 | 4h0gA | 0.61 | 0.60 | 0.98 | 3.40 |
| 4 | 4h0gA | 0.47 | 0.44 | 0.91 | 0.80 |
| 5 | 4hjjL | 0.42 | 0.39 | 0.90 | 0.84 |
| 6 | 1lmkA | 0.60 | 0.58 | 0.95 | 3.31 |
| 7 | 4hjjL | 0.41 | 0.39 | 0.90 | 1.29 |
| 8 | 2ghwB | 0.67 | 0.63 | 0.93 | 6.23 |
| 9 | 2ghwB | 0.66 | 0.63 | 0.93 | 2.56 |
| 10 | 2gkiA | 0.54 | 0.51 | 0.94 | 5.72 |

**Supplementary Table 4B.** Top 10 identified structural analogs of target HuscFv25 in PDB

| **Rank** | **PDB Hit** | **TM-score** | **RMDS^a^** | **IDEN^a^** | **Cov** |
| --- | --- | --- | --- | --- | --- |
| 1 | 4h0gA | 0.944 | 0.99 | 0.609 | 0.963 |
| 2 | 3juyB | 0.915 | 1.07 | 0.507 | 0.942 |
| 3 | 2ghwB | 0.910 | 0.89 | 0.668 | 0.930 |
| 4 | 3umtA | 0.904 | 0.85 | 0.482 | 0.922 |
| 5 | 3gm0A | 0.899 | 1.72 | 0.474 | 0.955 |
| 6 | 3uzvB | 0.898 | 1.26 | 0.504 | 0.938 |
| 7 | 4f9pD | 0.893 | 1.09 | 0.491 | 0.922 |
| 8 | 1qokA | 0.890 | 1.06 | 0.505 | 0.914 |
| 9 | 1dzbA | 0.882 | 1.30 | 0.486 | 0.914 |
| 10 | 4buhA | 0.877 | 1.32 | 0.473 | 0.914 |

**Supplementary Table 5A.** Top 10 threading templates used to model HuscFv34 by I-TASSER

| **Rank** | **PDB Hit** | **Iden1** | **Iden2** | **Cov** | **Norm. Z-score** |
| --- | --- | --- | --- | --- | --- |
| 1 | 2ghwB | 0.67 | 0.64 | 0.95 | 3.47 |
| 2 | 4h0gA | 0.56 | 0.55 | 0.98 | 4.42 |
| 3 | 4h0gA | 0.56 | 0.55 | 0.98 | 3.48 |
| 4 | 2ghwB | 0.67 | 0.64 | 0.95 | 0.76 |
| 5 | 4hjjL | 0.49 | 0.47 | 0.92 | 0.83 |
| 6 | 2ghwB | 0.67 | 0.64 | 0.95 | 3.32 |
| 7 | 4hjjL | 0.49 | 0.47 | 0.91 | 1.28 |
| 8 | 3umtA | 0.57 | 0.54 | 0.94 | 6.10 |
| 9 | 2ghwB | 0.67 | 0.64 | 0.95 | 2.57 |
| 10 | 2gkiA | 0.62 | 0.58 | 0.94 | 5.44 |

**Supplementary Table 5B.** Top 10 identified structural analogs of target HuscFv34 in PDB

| **Rank** | **PDB Hit** | **TM-score** | **RMDS^a^** | **IDEN^a^** | **Cov** |
| --- | --- | --- | --- | --- | --- |
| 1 | 4h0gA | 0.944 | 0.99 | 0.609 | 0.963 |
| 2 | 3juyB | 0.915 | 1.07 | 0.507 | 0.942 |
| 3 | 2ghwB | 0.910 | 0.89 | 0.668 | 0.930 |
| 4 | 3umtA | 0.904 | 0.85 | 0.482 | 0.922 |
| 5 | 3gm0A | 0.899 | 1.72 | 0.474 | 0.955 |
| 6 | 4f9pD | 0.898 | 1.26 | 0.504 | 0.938 |
| 7 | 1qokA | 0.893 | 1.09 | 0.491 | 0.922 |
| 8 | 3uzvB | 0.890 | 1.06 | 0.505 | 0.914 |
| 9 | 1dzbA | 0.882 | 1.30 | 0.486 | 0.914 |
| 10 | 4buhA | 0.877 | 1.32 | 0.473 | 0.914 |

**Supplementary Table 6.** Qualities of the I-TASSER predicted HuscFv models used in this study.

| Name | C-score | Exp.TM-Score | Exp.RMSD (Å) | No. of decoys | Cluster density |
| --- | --- | --- | --- | --- | --- |
| HuscFv6 | 1.11 | 0.87 ± 0.07 | 3.5 ± 2.4 | 9859 | 0.7435 |
| HuscFv10 | 1.15 | 0.87 ± 0.07 | 3.5 ± 2.4 | 9395 | 0.7676 |
| HuscFv25 | 0.96 | 0.87 ± 0.08 | 3.8 ± 2.6 | 9511 | 0.6475 |
| HuscFv34 | 1.02 | 0.85 ± 0.08 | 3.7 ± 2.5 | 9532 | 0.6821 |

**C-score,** a confidence score for estimating the quality of I-TASSER predicted model calculated based on the significance of threading template alignments and the convergence parameters of the structure assembly simulation. C-score is typically in the range of -5 to 2, where a C-score of higher value signifies a model with a high confidence and *vice-versa.*

**TM-score and RMSD,** the known standards for measuring structural similarity between two structures which are used usually to measure the accuracy of modeled structure when the native structure is known. In the case which the native structure is not known, it becomes necessary to predict the quality of the model prediction.
