# Supplementary material for: Human Transbodies to HCV NS3/4A Protease Inhibit Viral Replication and Restore Host Innate Immunity
Source: Front Immunol. 2016 Aug 26;7:318. doi: 10.3389/fimmu.2016.00318 (PMC4999588; doi:10.3389/fimmu.2016.00318)

Supplementary Figure 1 Appearance of the HCV foci in cells of individual treatment groups after staining with anti-HCV core protein as the primary antibody. A-I, transfected cells treated with R9-HuscFv6, R9-HuscFv10, R9-HuscFv25, R9-HuscFv34, Peg-IFN + ribavirin, telaprevir, Peg-IFN + ribavirin + telaprevir, control R9-HuscFv, and medium alone. J, non-transfected cells.

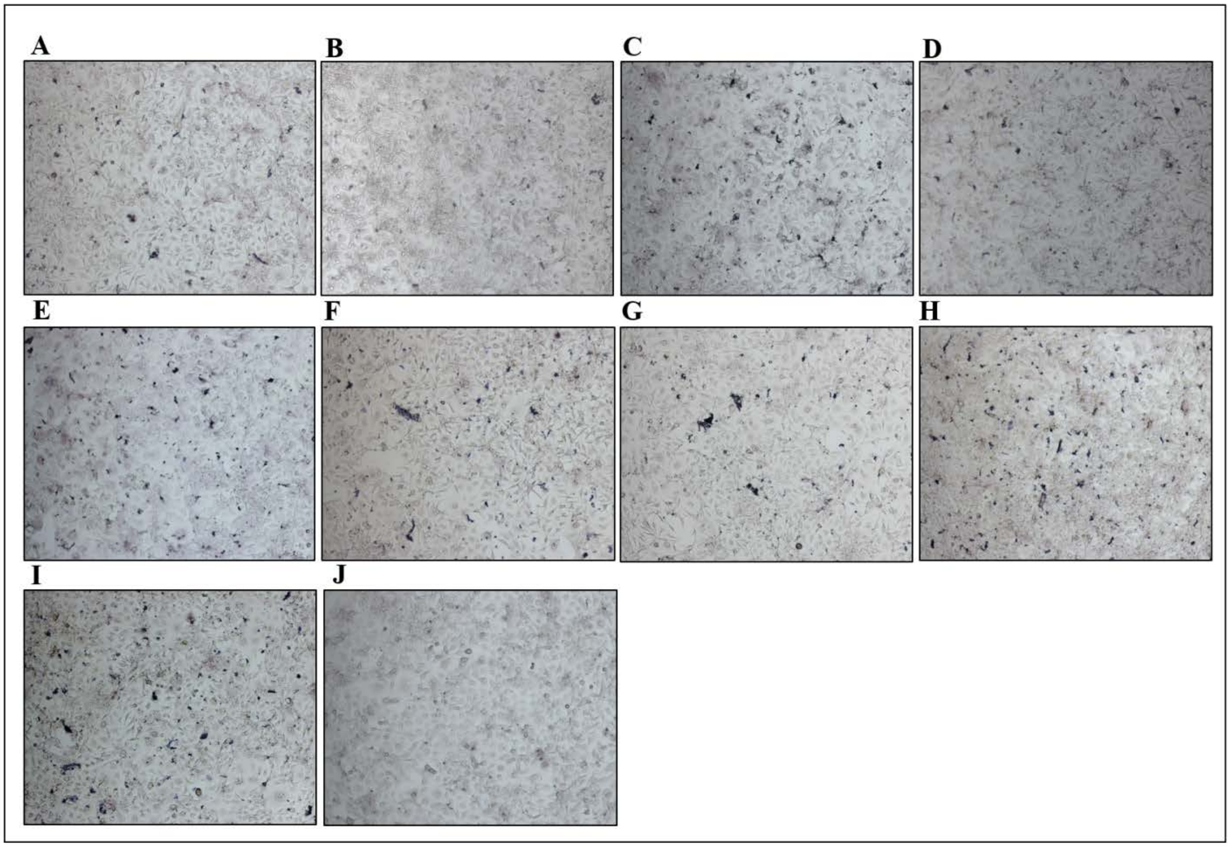

Supplement: Supplementary file 3 [file Image_1.PDF]
