# Supplementary figures and images for: Human Transbodies to HCV NS3/4A Protease Inhibit Viral Replication and Restore Host Innate Immunity
Source: Front Immunol. 2016 Aug 26;7:318. doi: 10.3389/fimmu.2016.00318 (PMC4999588; doi:10.3389/fimmu.2016.00318)

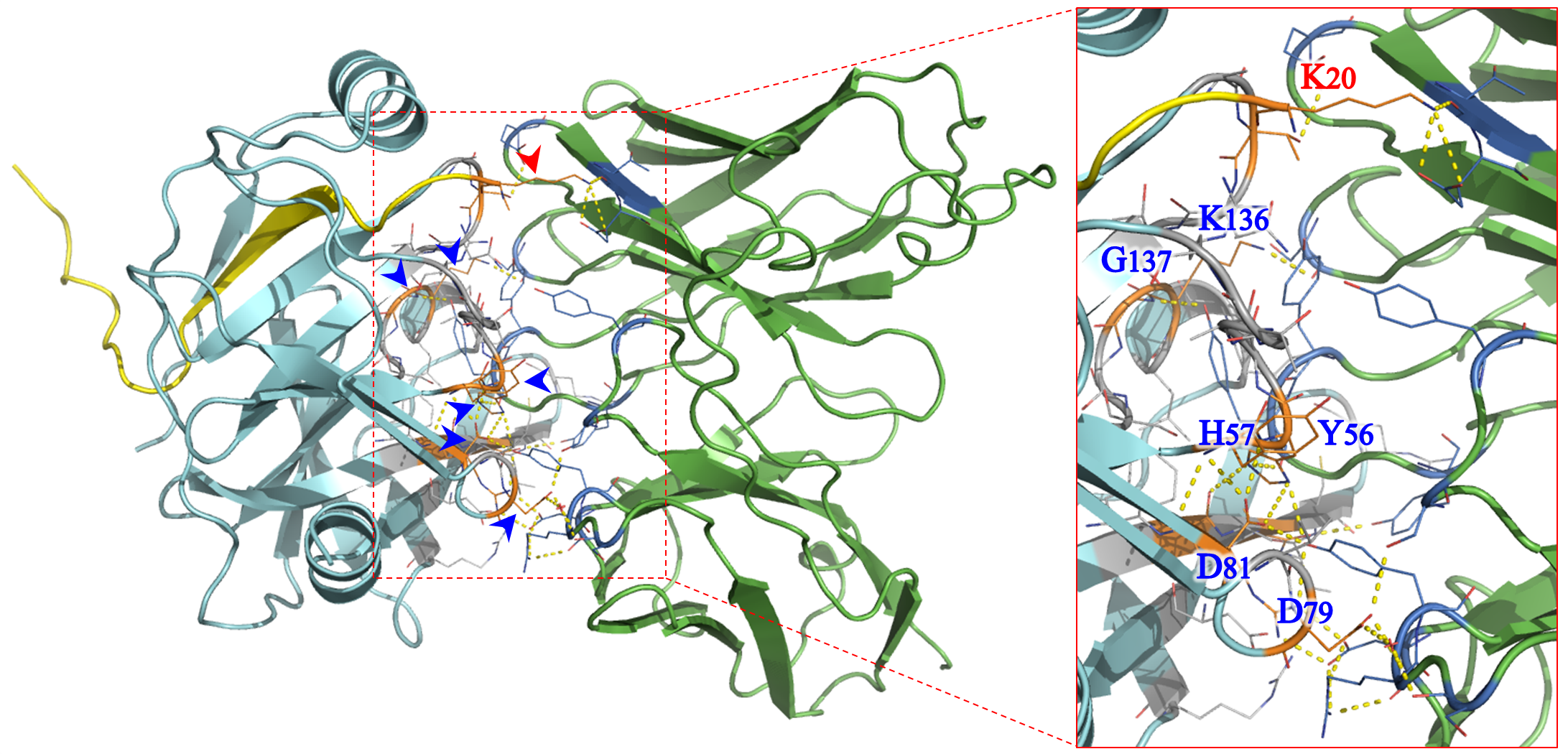

Supplement: Supplementary file 4 [file Image_2.TIF]

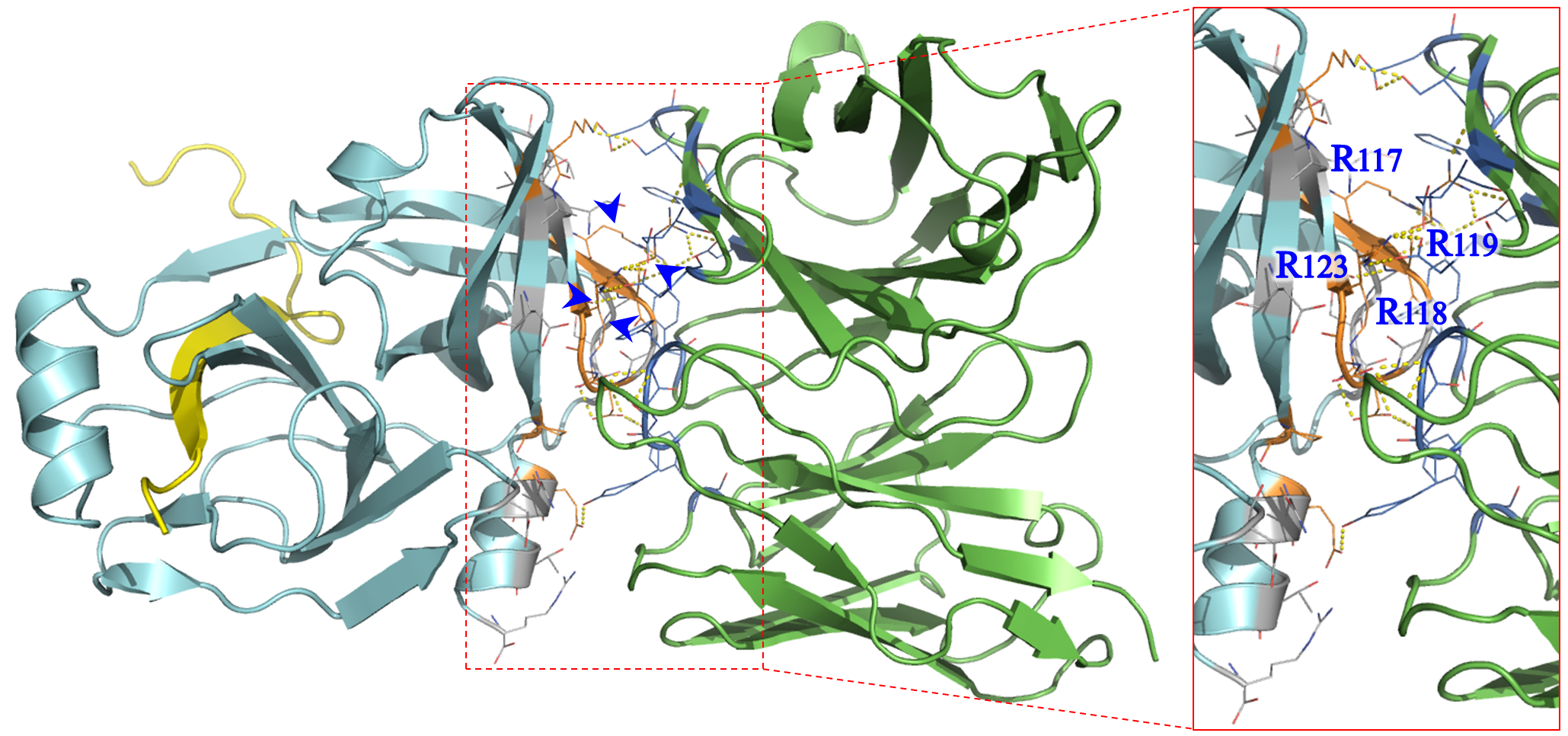

Supplement: Supplementary file 5 [file Image_3.TIF]

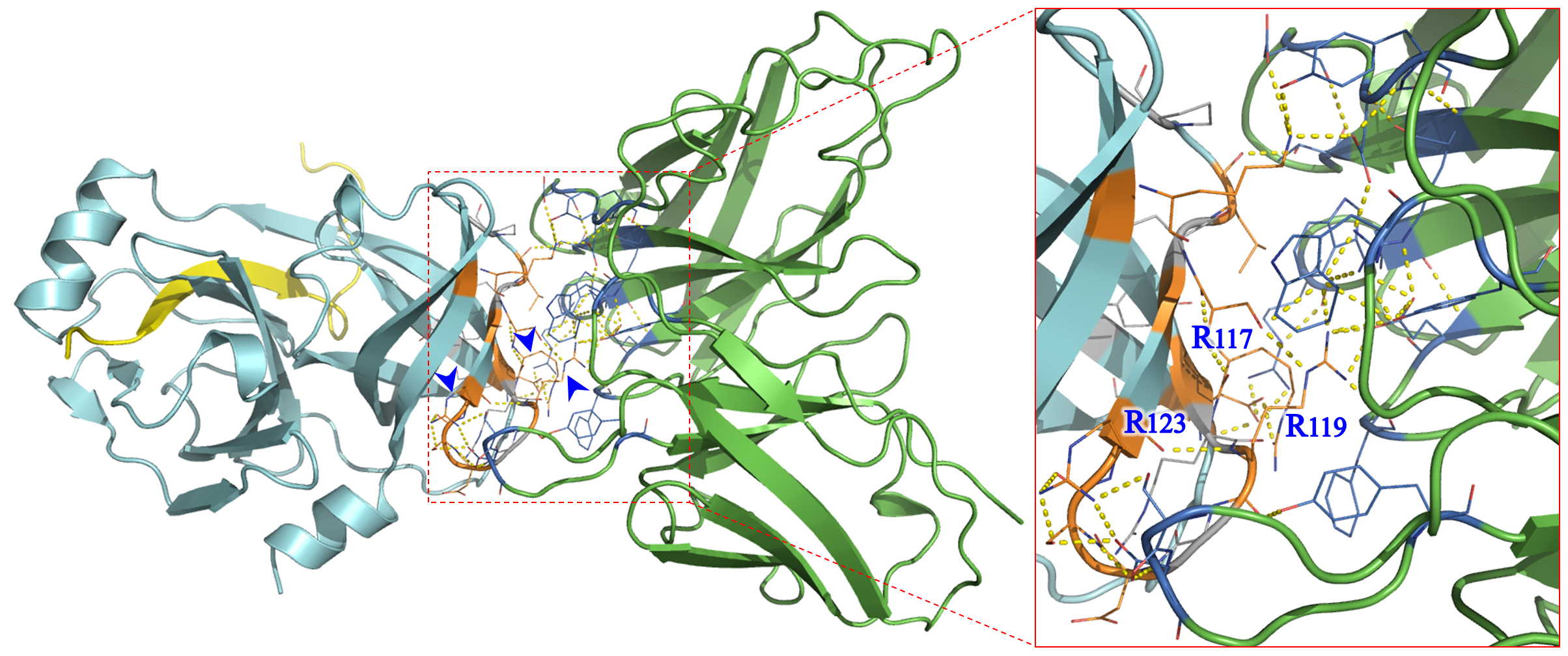

Supplement: Supplementary file 6 [file Image_4.TIF]

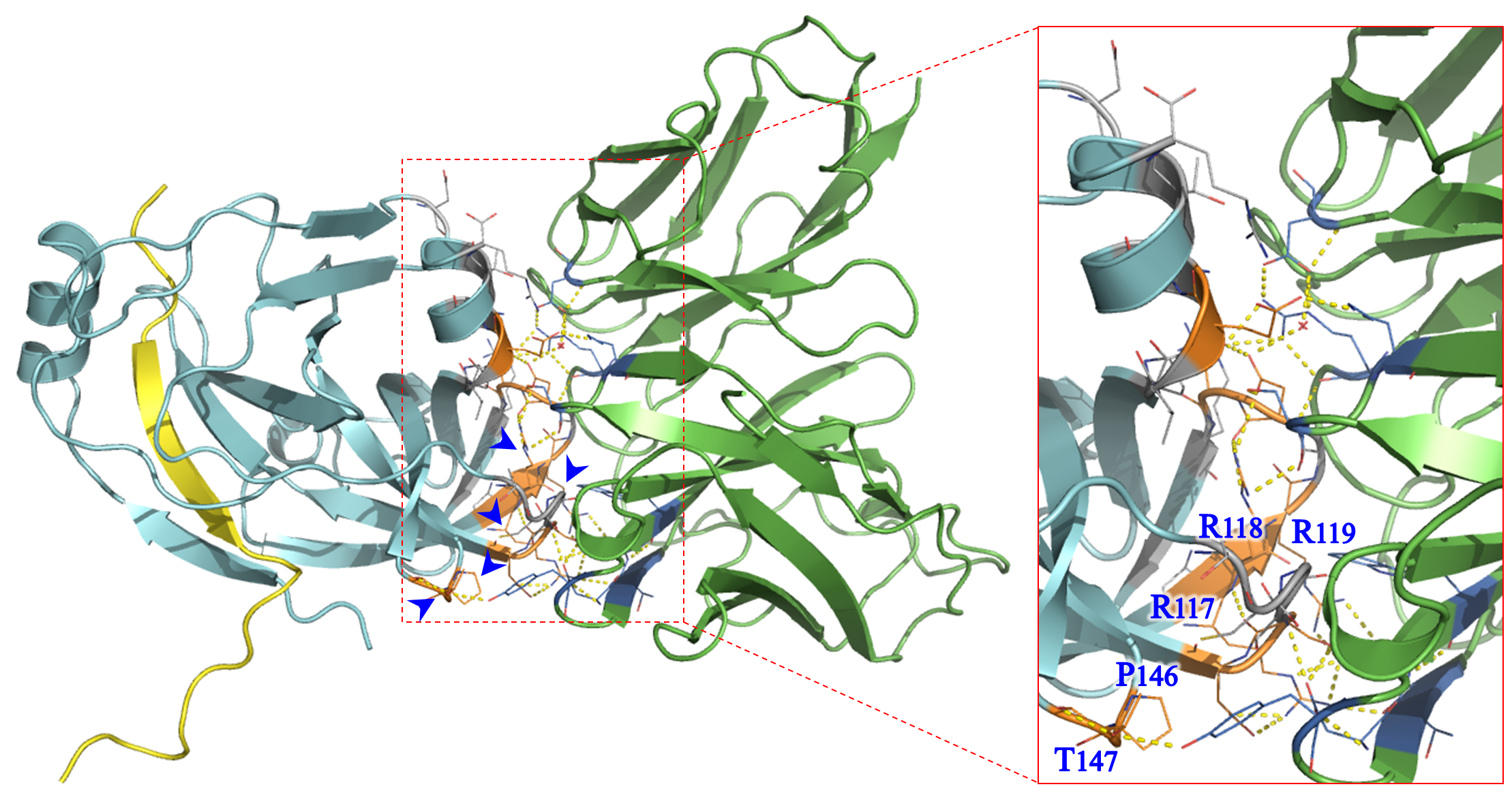

Supplement: Supplementary file 7 [file Image_5.TIF]
